# Oxytocin for Male Subjects with Autism Spectrum Disorder and Comorbid Intellectual Disabilities: A Randomized Pilot Study

**DOI:** 10.3389/fpsyt.2016.00002

**Published:** 2016-01-21

**Authors:** Toshio Munesue, Hiroyuki Nakamura, Mitsuru Kikuchi, Yui Miura, Noriyuki Takeuchi, Tokie Anme, Eiji Nanba, Kaori Adachi, Kiyotaka Tsubouchi, Yoshimichi Sai, Ken-ichi Miyamoto, Shin-ichi Horike, Shigeru Yokoyama, Hideo Nakatani, Yo Niida, Hirotaka Kosaka, Yoshio Minabe, Haruhiro Higashida

**Affiliations:** ^1^Research Center for Child Mental Development, Kanazawa University, Kanazawa, Japan; ^2^Department of Environmental and Preventive Medicine, Graduate School of Medical Sciences, Kanazawa University, Kanazawa, Japan; ^3^International Community Care and Lifespan Development, Empowerment Sciences, Faculty of Medicine, University of Tsukuba, Tsukuba, Japan; ^4^Division of Functional Genomics, Research Center for Bioscience and Technology, Tottori University, Yonago, Japan; ^5^Department of Pharmacy, Kanazawa University Hospital, Kanazawa, Japan; ^6^Advanced Science Research Center, Kanazawa University, Kanazawa, Japan; ^7^Department of Psychiatry and Neurobiology, Graduate School of Medical Sciences, Kanazawa University, Kanazawa, Japan; ^8^Division of Genomic Medicine, Department of Advanced Medicine, Medical Research Institute, Kanazawa Medical University, Uchinada, Japan; ^9^Research Center for Child Mental Development, University of Fukui, Fukui, Japan

**Keywords:** autism spectrum disorder, Kanner type, oxytocin, intranasal administration, social behavior

## Abstract

Approximately half of autism spectrum disorder (ASD) individuals suffer from comorbid intellectual disabilities (IDs). Oxytocin (OXT) receptors are highly expressed in temporal lobe structures and are likely to play a modulatory role in excitatory/inhibitory balance, at least based on animal model findings. Thus, it is feasible that in the highly representative group of Kanner-type ASD subjects, OXT could have a beneficial effect on social communication and social interaction. The aim of this pilot study is to investigate the feasibility and adverse events, such as epilepsy, of the long-term administration of intranasal OXT for adolescent and adult ASD subjects with ID because such patients frequently have seizures. We also addressed the question on how to scale the OXT effects to the core symptoms of social deficits because of the relative difficulty in obtaining objective measurements. Twenty-nine males (aged 15–40 years old) participated in a randomized, double-blind, and placebo-controlled crossover study (each for 8 weeks) with OXT (16 IU/day). Except for seizures experienced by one participant, other serious adverse events did not occur. The primary and secondary outcomes measured using the Childhood Autism Rating Scale and several standard scales, respectively, revealed no difference between the OXT and placebo groups. Instead, in an exploratory analysis, the social interactions observed in the play sessions or in daily life were significantly more frequent in the initial half period in the OXT-first arm of the crossover trial. There were also significant correlations between the plasma OXT concentration and subscale scores for irritability on the Aberrant Behavior Checklist. In conclusion, this pilot study demonstrates that long-term administration of intranasal OXT is tolerable in a representative cohort of ASD individuals with ID and suggests that future multicenter trials of OXT are warranted and should include measurements of reciprocal social interactions based on daily life under closer surveillance for epilepsy. Trial registration: UMIN000007250.

## Introduction

Autism spectrum disorder (ASD) is a neurodevelopmental and life-long syndrome with multiple etiologies, different phenotypes, and a high prevalence, estimated at 0.62–2% (1–3). The core symptoms of ASD have consisted of social and communication impairments and repetitive behaviors since the earliest reports by Leo Kanner in 1943 and Hans Asperger in 1944 (4–6). The clinical domain of social deficits, expressed symbolically as “autism,” results in serious maladjustment in individuals with ASD in their daily lives (7). The societal domain of social deficits, conceptualized as theory of mind impairments (8), addresses critically interdisciplinary issues, including social psychology (9) and philosophy (10). In clinical settings, there are no effective treatments for the various symptoms of ASD, except for irritability, which can be alleviated by risperidone (11) or aripiprazole (12). As for social deficits, little to no pharmacological treatments have been proven to be effective (13, 14), although recent studies suggest the efficacy of risperidone for social disabilities based on the Aberrant Behavior Checklist (ABC) subscale of social withdrawal (15, 16) and the promising candidate neuroendocrine hormone oxytocin [OXT (17, 18)].

The results of four clinical trials on short-term OXT administration performed over the last decade (19–22) and a meta-analysis of a single administration suggest that OXT has significant benefits compared with a placebo in the treatment of ASD, although it has a moderate effect size (Cohen’s *d* = 0.57) (23). Notably, social deficits have been reported to be relieved by OXT administration (20–22, 24–26). Over the last 5 years, nearly 20 clinical trials have been registered to investigate whether long-term OXT treatment is beneficial. Among them, two recent studies have yielded inconsistent results: in one study, while no effect was found on the primary outcome, the three secondary outcomes were significantly improved by OXT (27); by contrast, in the other report, none of the measures showed any significant differences between OXT and the placebo (28). Interestingly, the “reading the mind in the eyes” test revealed significantly favorable responses in the social deficits of ASD subjects following OXT administration (27).

Approximately 56% of ASD individuals suffer from a comorbid intellectual disability (ID) (29). In addition, the prevalence of epilepsy has been estimated to be 46% in ASD patients with low intelligence quotient (IQ) (30). Because these patients have rarely been subjects in recent biological and clinical studies (31), whether they would receive benefits from OXT remains unknown. In addition, OXT receptors are highly expressed in the temporal lobe of the brain and are likely to play a modulatory role in maintaining the excitatory/inhibitory balance (32), at least based on results of animal model studies (32, 33). Thus, it is feasible that in the highly representative group of Kanner-type ASD subjects, OXT could have a beneficial effect on social communication and interaction. Therefore, an OXT clinical trial in this important population of individuals with the so-called Kanner type of ASD is warranted (5). Here, our first aim is to investigate the potential therapeutic effects of intranasal OXT administration on a variety of symptoms or adverse effects, including seizures, in adolescents and adults with ASD and a comorbid ID. Another aim is to determine which measurements are applicable for such individuals because it is relatively difficult to obtain objective measurements using computer tests and brain imaging instruments in which the patients have to correctly respond to instructions during examinations. To address this difficulty, we conducted an exploratory analysis of daily-life behaviors from a list of real-life events of reciprocal social interactions that were obtained from the patients’ caregivers and an involved doctor, in addition to primary and secondary outcomes measured by existing standard scales that are typically applied to ASD patients. We also measured the plasma OXT concentration of ASD participants with ID to examine the relationship between the OXT concentration and behavioral indexes.

## Materials and Methods

The CONSORT checklist and protocol for this study are available as Datasheets S1 and S2 in Supplementary Material, respectively.

### Ethics Statement

This study was approved by the institutional review board in the Innovative Clinical Research Center of Kanazawa University Hospital, Japan [Approval No. 2011-045 (5749)]. The study was performed according to the Declaration of Helsinki and the Ethical Guidelines for Clinical Studies of the Ministry of Health, Labour, and Welfare of Japan. Following a complete explanation of the study, all of the caregivers of the participants provided written informed consent, and participants provided written informed assent if they could read and understand the informed assent form (Datasheet S3 in Supplementary Material), which we composed in concise and understandable language.

### Study Design and Setting

We designed a pilot, randomized, double-blind, and placebo-controlled crossover study conducted from February 2012 to October 2013. The study was registered with the University Hospital Medical Information Network (UMIN) Clinical Trials Registry (number UMIN000007250; https://upload.umin.ac.jp/cgi-open-bin/ctr/ctr.cgi?function=search&action=input&language=E). The study was conducted in the outpatient setting of the Department of Child and Adolescent Psychiatry of Kanazawa University Hospital in Kanazawa, Japan.

The two treatment periods were combined into one period (16 weeks) by setting the beginning of the second treatment period as the baseline. During the initial study design, we thought that crossover would provide complete results; we expected the effect of OXT would appear in the OXT arm and not in the placebo arm. In the second term, we again expected that the OXT effect would appear in the OXT arm in which little-to-no effects were observed during the initial placebo treatment and that the OXT effect would disappear gradually in the placebo period. Since no information was available for setting the length of the washout period in 2012, the two phases were conducted consecutively without the washout period.

### Participants

Eligible participants were male adolescents and adults aged 15–45 years. They had been diagnosed with a pervasive developmental disorder, as defined by the Diagnostic and Statistical Manual of Mental Disorders, fourth edition, text revision (DSM-IV-TR) (34). The diagnoses of all of the participants were confirmed using the Diagnostic Interview for Social and Communication Disorders, eleventh edition (35), by the first author, who is certified. All of the participants were required to have an ID measured as an IQ < 70 on the Tanaka–Binet Intelligence Scale [a Japanese version of the Stanford–Binet Intelligence Scale (36)].

The exclusion criteria included a concomitant or previous history of severe polydipsia, water intoxication (37–39) or substance dependence, or a previous history of traumatic brain injury with a loss of consciousness for more than 5 min.

We have previously observed behaviorally favorable changes in several outpatients with ASD and a comorbid ID who received over-the-counter OXT (40). We estimated that approximately 60% of these ASD patients benefited from OXT. Accordingly, we tentatively estimated a sample size of 30 in a crossover design. We expected that we would be able to recruit 30 participants among 150 or more patients with ASD who regularly visited the Kanazawa University Hospital to receive drug therapies or psychosocial interventions. Fourteen candidates were recruited from a clinics specializing in ASD and the University of Fukui Hospital in neighboring towns.

### Randomization and Blinding

**Figure 1:**
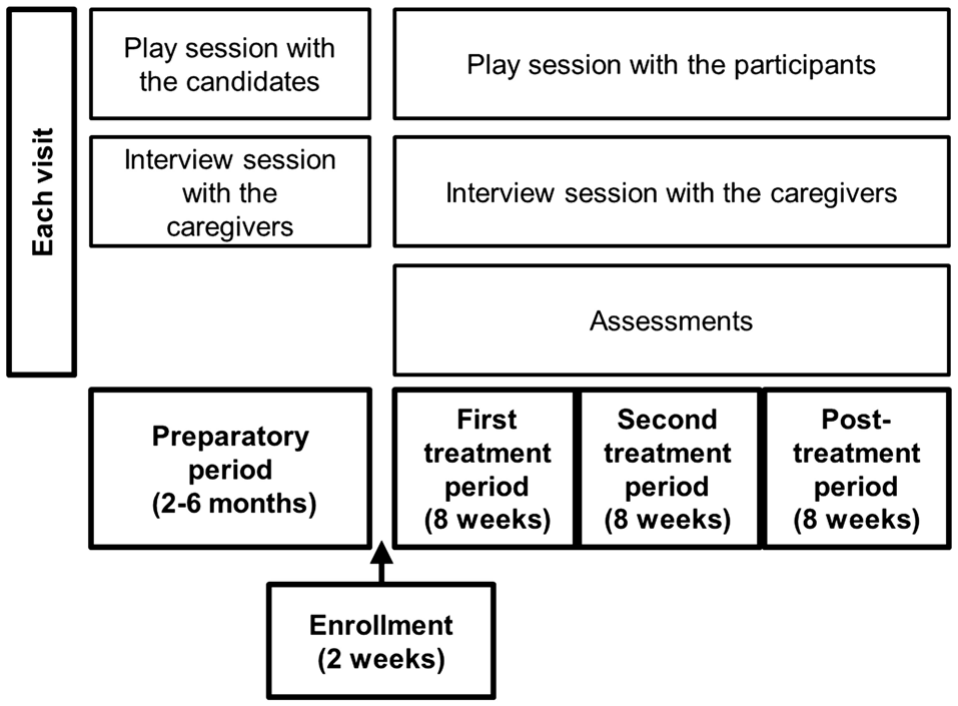
**Schema of the study**. Prior to study enrollment, participants, and their caregivers visited Kanazawa University Hospital every 2 weeks during the preparatory period. Each visit consisted of a play session with a participant and an interview session with the caregivers. After the study began, the participants and caregivers visited the hospital eight times every 2 weeks and engaged in the same activities.

Eligible participants completed baseline evaluations and were randomly assigned at a 1:1 ratio to receive either 8 IU of OXT twice per day or a matching placebo twice per day for 8 weeks during the first treatment period (Figure 1). The participants were then crossed over, without washout, to the other treatment for 8 weeks during the second treatment period. The two treatment periods were followed by an 8-week posttreatment period without treatment administration. We adopted a crossover design because of ethical considerations. Randomization was centralized, and a computer-generated list with random block sizes of six was used in the Innovative Clinical Research Center, Kanazawa University Hospital. The study staff, participants, and their caregivers did not have access to the allocation sequence until the end of the study.

### Treatments

Nasal spray bottles were prepared by two pharmacists in the Kanazawa University Hospital. Syntocinon (Novartis, Huningue, France; 5 ml per bottle) in the original bottles was transferred to sterile nasal spray bottles (Oono, Co., Ltd., Tokyo, Japan). One puff of the bottle was confirmed to contain 4 IU of OXT. An identical bottle was prepared containing 5 ml of placebo, consisting of all of the ingredients in Syntocinon, except OXT. The participants practiced puffing the placebo into their nostrils with the assistance of their caregivers for 2–4 weeks at their homes prior to the commencement of the study.

### Play Sessions

Prior to the study, 20-min play sessions were held between each subject and the first author every 2 weeks for 2–6 months in the playroom of our hospital (the preparatory period; Figure 1). We aimed for the ASD participants with ID to become familiar with the study setting, electrocardiography, and blood collection in the hospital during these prestudy sessions. We also carefully observed the participants’ behaviors.

Each participant visited the hospital every 2 weeks during the study. Each visit included a 20-min play session, which was recorded by two video cameras set up diagonally in the playroom, and an interview session with the caregivers (Figure 1). During the play sessions, the first author sat or stood close to each participant, observed him, and sometimes addressed him or showed him toys. The first author did not pressure a social interaction with the participants; instead, he took a passive role to be spontaneously approached by the participant.

### Outcomes

The treatment adherence rate was ascertained by a treatment diary in which the spraying times in the morning and evening were recorded by the caregivers.

### Assessment of Harm

The caregivers reported all of the events using a questionnaire developed for this study at each visit (Datasheet S4 in Supplementary Material). We measured the body weight, blood pressure, and pulse rate every 4 weeks. Blood work, urinalysis, and electrocardiography were performed every 4 weeks. The blood work included assessments of the blood counts, electrolytes, renal and liver function, and osmolality. Urine osmolality was measured in addition to routine urinalysis assessments. Although these measurements would have been informative, the participants were not monitored with electroencephalography, magnetoencephalography, magnetic resonance imaging, and computed tomography because it was difficult to attach probes to the patients’ heads and to get them to lie down on the bed without movement.

### Primary Outcome Measure

A change in the primary outcome was evaluated using the Childhood Autism Rating Scale (CARS) (41).

### Secondary Outcome Measures

The secondary outcomes consisted of changes in scores evaluated using the ABC (16), the Clinical Global Impressions – Improvement (CGI-I) scale, and the Global Assessment of Functioning (GAF) (34, 42). The Interaction Rating Scale Advanced (IRSA) (43, 44) was used for measuring six domains in social interaction. The patients’ social behavior was assessed by inspecting the videos captured by two fixed-angle cameras in the playroom in the Kanazawa University Hospital. As for CGI-I, we regarded a participant as a responder only when he was categorized as clinically improved (CGI-I ≤ 2).

### Exploratory Outcomes: Real-Life Assessments of Social Interactions

From the beginning of this study, we noticed the necessity of a special analysis for changes in outcomes of social deficits as a core symptom of ASD and in plasma OXT levels in our patients with ID. The possibility of an assessment of social interaction based on medical charts had been included in the original protocol (lines 8–17 in page 16 in Datasheet S2 in Supplementary Material), although we did not describe the exact methods to analyze the OXT effects on social interaction and the OXT concentrations. However, we decided to use the recordings in the medical chart after 1 month from start of statistical analyses because details of the participants’ behaviors obtained during an interview session with the caregivers and during the play sessions with participants were entered into the medical charts by the first author. Details of the participants’ behaviors in their daily life since the last visit were freely recounted by caregivers during the interview sessions and were entered into the medical charts by the first author (Figure S1 in Supplementary Material). These descriptions included various episodes that had not been previously observed for the participants. For example, a mother reported the following: “My son (40 years old with profound ID) pours hot Japanese tea in a teacup from a teapot for me upon my request. Usually, he leaves the teacup standing. The other day, however, further *handed* it to me. That was his first behavioral change. I was very happy by this.” These episodes may merely refer to unintentional observations by the first author or free statements of recollection by caregivers. Moreover, the documents recorded by the first author varied in the amount and quality for each participant and for each visit. However, we believe that there may be meaningful episodes, such as the above example, in these fragmentary descriptions.

After this clinical trial and observation was completed, all data, including the medical charts, were fixed and controlled by the clinical trial control center. After competing of analyses of primary and secondary outcomes (except for IRSA), social interaction from the medical chart were selected by the two coauthors (Noriyuki Takeuchi, a sociologist, and Yui Miura, a psychologist). They were involved only in the data analysis of this study and never met the participants or their caregivers. The two researchers inspected and examined the all documents for 29 participants that were anonymously printed from the medical chart of each participant for 3 days in the first week and repeated twice in the following week. Subsequently, they independently sampled episodes that were identified as reciprocal social interactions. The definition of “reciprocal social interaction” refers to descriptions determined as indicating the existence of interpersonal exchanges between a participant and the first author or caregivers. We counted these episodes at each visit and then scored them dichotomously as plus (number ≥ 1) or minus (number = 0) an index of real-life social interactions at each visit. The intrarater reliability was tested three times with >90% reliability, and interrater reliability was approximately 85%. Reliability was calculated by the Fisher’s exact probability test. We then used these data as exploratory outcomes.

### Plasma OXT Concentrations

The OXT immunoreactivity levels on unextracted samples were quantified by an OXT enzyme immunoassay kit (Catalog No. ADI-900-153, Assays Designs, MI, USA) as previously described (40, 45). Blood was collected between 2 and 5 p.m. every 4 weeks. The participants were instructed to abstain from food for 2 h prior to blood collection.

### Evaluators of Each Outcome

The scores on the CARS, the CGI-I scale, and the GAF and adverse events and treatment adherence were evaluated by the first author. The ABC was noted by the caregivers. The IRSA was assessed by a coauthor (Tokie Anme) using videotaped images. Real-life assessments of social interactions were rated by the two coauthors (Noriyuki Takeuchi and Yui Miura). Figure S1 in Supplementary Material presents the interval of each assessment. The plasma OXT concentration was assayed by a coauthor (Haruhiro Higashida).

### Statistical Analysis

The comparisons of interest were the sequential differences between the two treatment groups in seven measurements. In our crossover study, we employed a generalized mixed regression analysis, with treatment (OXT or placebo), time (each visit), and order (OXT then placebo or placebo then OXT) as the independent factors. The two treatment periods were combined into one period (8 weeks) by setting the beginning of the second treatment period as the baseline. When data of variables in participant characteristics were significantly different between the OXT-first group and the placebo-first group, the variables were included as covariates in the analysis. When a significant order effect was identified, additional generalized mixed regression analyses with treatment (OXT or placebo) and time (each visit) as the independent factors were applied for only the first treatment period. All of the statistical analyses were performed using Statistical Package for the Social Sciences (SPSS) software, version 21 (IBM Japan, Tokyo, Japan). We planned this pilot study to show the feasibility of conducting a larger study in the future and to collect data to help optimize its design. Because of the small sample size (i.e., low statistical power), the significance level was set at 0.05, and we did not employ correction for multiple comparisons.

## Results

**Figure 2:**
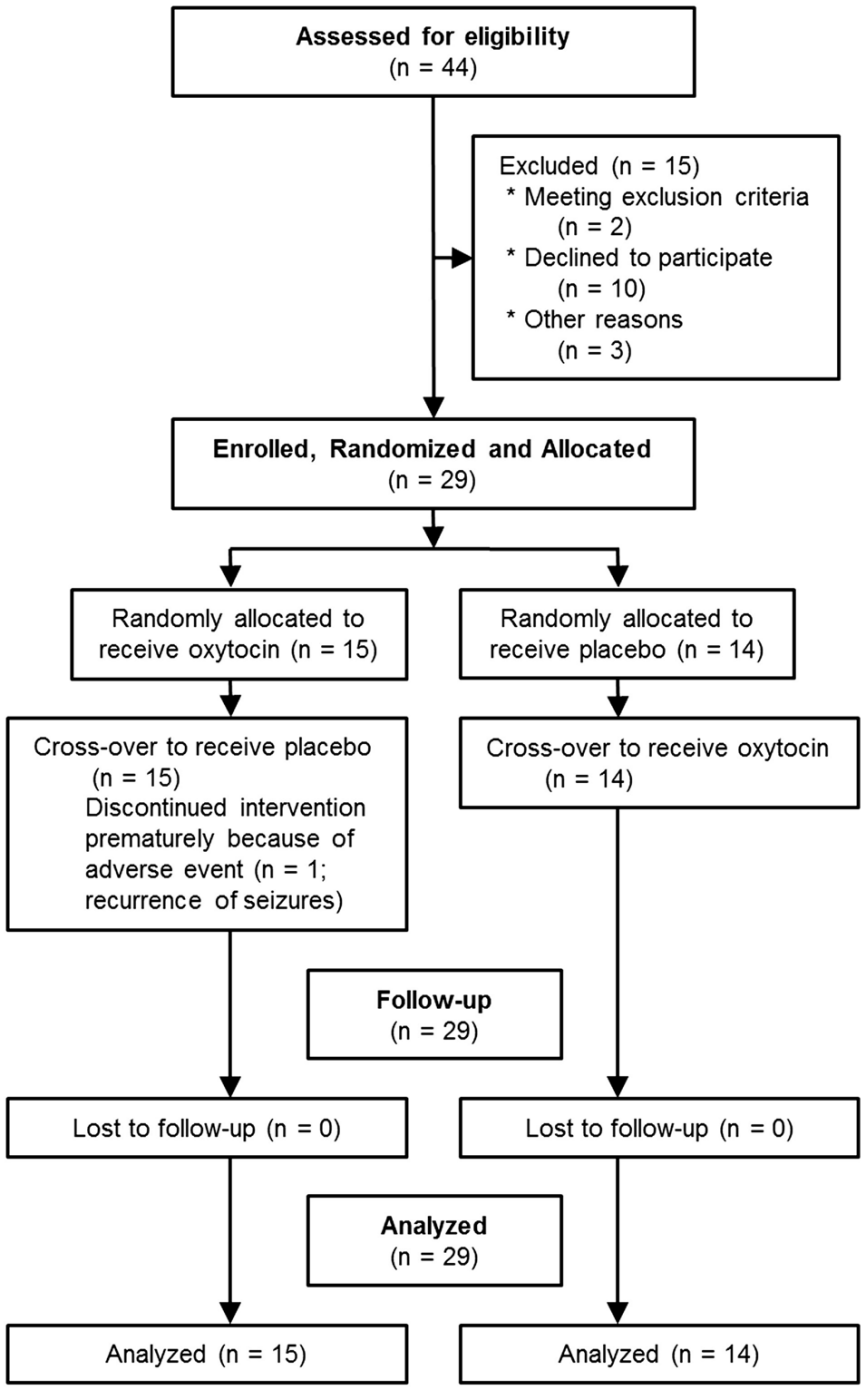
**CONSORT 2010 flow diagram**.

As shown in Figure 2, 15 patients were excluded from the initial 44 patients recruited. Thus, we enrolled 29 participants, and 28 were able to complete the study. One participant experienced seizures during the first and second treatment periods and discontinued the study in the second period.

Table 1 summarizes the baseline characteristics of the allocated participants. All of the participants were diagnosed as having autistic disorder according to the DSM-IV-TR. No significant differences were found between the two groups with regards to age, psychotropic medications, or epilepsy. The participants were aged 15–40 years (mean ± SD = 22.5 ± 5.9) and had IQs ranging from immeasurable to 59 (mean ± SD = 32.6 ± 14.7). None of the participants had histories of common medical conditions associated with syndromic ASD, such as tuberous sclerosis. Seven participants (24.1%) had comorbid stable epilepsy, and one subject suffered from recurrent attacks once or twice per year. Twenty-two participants (75.9%) received psychotropic medications at stable doses during the 3 weeks prior to randomization. We did not observe any significant differences in the behavioral assessments using the CARS, ABC, GAF, and IRSA between the two groups (Table 1). However, the proportion of the severity of ID was significantly different between the OXT-first and the placebo-first groups (*P* = 0.013, Fisher’s exact test). We included the variable as covariate in the following analysis. More detailed characteristics of each participant are presented in Table S1 in Supplementary Material.

**Table 1:** **Participant characteristics by treatment arm**.

Characteristics	Oxytocin first	Placebo first	All participants
	
	(*N* = 15)	(*N* = 14)	(*N* = 29)
Age (years; mean ± SD)	22.6 ± 4.3	22.4 ± 7.4	22.5 ± 5.9
Intelligence quotient			
Measurable (mean ± SD)	24.9 ± 13.2	37.5 ± 13.9	32.6 ± 14.7
Immeasurable	8 (53.3%)	3 (21.4%)	11 (37.9%)
Intellectual disabilities			
Mild	1 (6.7%)	2 (14.3%)	3 (10.3%)
Moderate	0 (0%)	6 (42.9%)	6 (20.7%)
Severe	3 (20%)	1 (7.1%)	4 (13.8%)
Profound	11 (73.3%)	5 (35.7%)	16 (55.2%)
Psychotropic medications	12 (80%)	10 (71.4%)	22 (75.9%)
Epilepsy	4 (26.7%)	3 (21.4%)	7 (24.1%)
Behavioral assessments			
CARS (mean ± SD)	43.1 ± 3.0	41.4 ± 4.5	42.3 ± 3.8
ABC (mean ± SD)			
Irritability	11.9 ± 9.4	17.4 ± 11.7	14.6 ± 10.7
Lethargy	12.8 ± 11.1	12.6 ± 6.1	12.7 ± 8.9
Stereotypic behavior	7 ± 5.9	5.2 ± 5.8	6.1 ± 5.8
Hyperactivity	13.9 ± 9.4	16 ± 10.3	14.9 ± 9.7
Inappropriate speech	4.9 ± 3.0	7 ± 3.7	5.9 ± 3.5
Total	50.3 ± 28.0	58.3 ± 26.8	54.2 ± 27.2
GAF (mean ± SD)	31.3 ± 6.0	35.6 ± 9.7	33.5 ± 8.1
IRSA (mean ± SD)	165.6 ± 28.8	189.1 ± 32.6	177.3 ± 32.5
Plasma OXT concentration (pg/ml; mean ± SD)	217.6 ± 165.2	306.3 ± 341.3	263.6 ± 270.0

*CARS, Childhood Autism Rating Scale; ABC, Aberrant Behavior Checklist; GAF, Global Assessment of Functioning; IRSA, Interaction Rating Scale Advanced; OXT, oxytocin*.

*Details for all of the participants are summarized in Table S1 in Supplementary Material*.

The mean values of treatment adherence ranged from 96.3 to 99.7% in the OXT-first group and from 97.1 to 99.5% in the placebo-first group during the 16-week treatment period (Table S2 in Supplementary Material). The intranasal sprayings were administered immediately after breakfast and dinner to all participants.

### Assessment of Harms

All of the adverse events reported by family members according to the questionnaire are listed in Table S3 in Supplementary Material. There were no differences in the number of adverse events between the OXT and placebo arms. No significant differences occurred in body weight, diastolic blood pressure, systolic blood pressure, and pulse rate between the two groups (Table S4 in Supplementary Material). No difference was observed between the results of the blood work and urinalysis or electrocardiography of the two groups (data not shown).

One participant (22 years old) experienced a seizure on the 33rd day of the first OXT treatment period. On the eighth day of the second treatment period, he had another seizure and discontinued the study, although he had received antiepileptic medications from the age of 5 years old and had not suffered a seizure since the age of 15 years old. His treatment adherence to OXT and antiepileptics was excellent. He did not exhibit hyponatremia during the study. Thereafter, seizures occurred repeatedly two or three times a month, although the doses of his antiepileptics had been increased. Another participant with stable epilepsy experienced a seizure on the 36th day of the posttreatment period. This participant was allocated to receive OXT administration in the second treatment period. We postulate that this seizure could be attributed to his forgetting to take antiepileptic medication for 2 or 3 days prior to the seizure.

### Primary Outcome Measure

**Figure 3:**
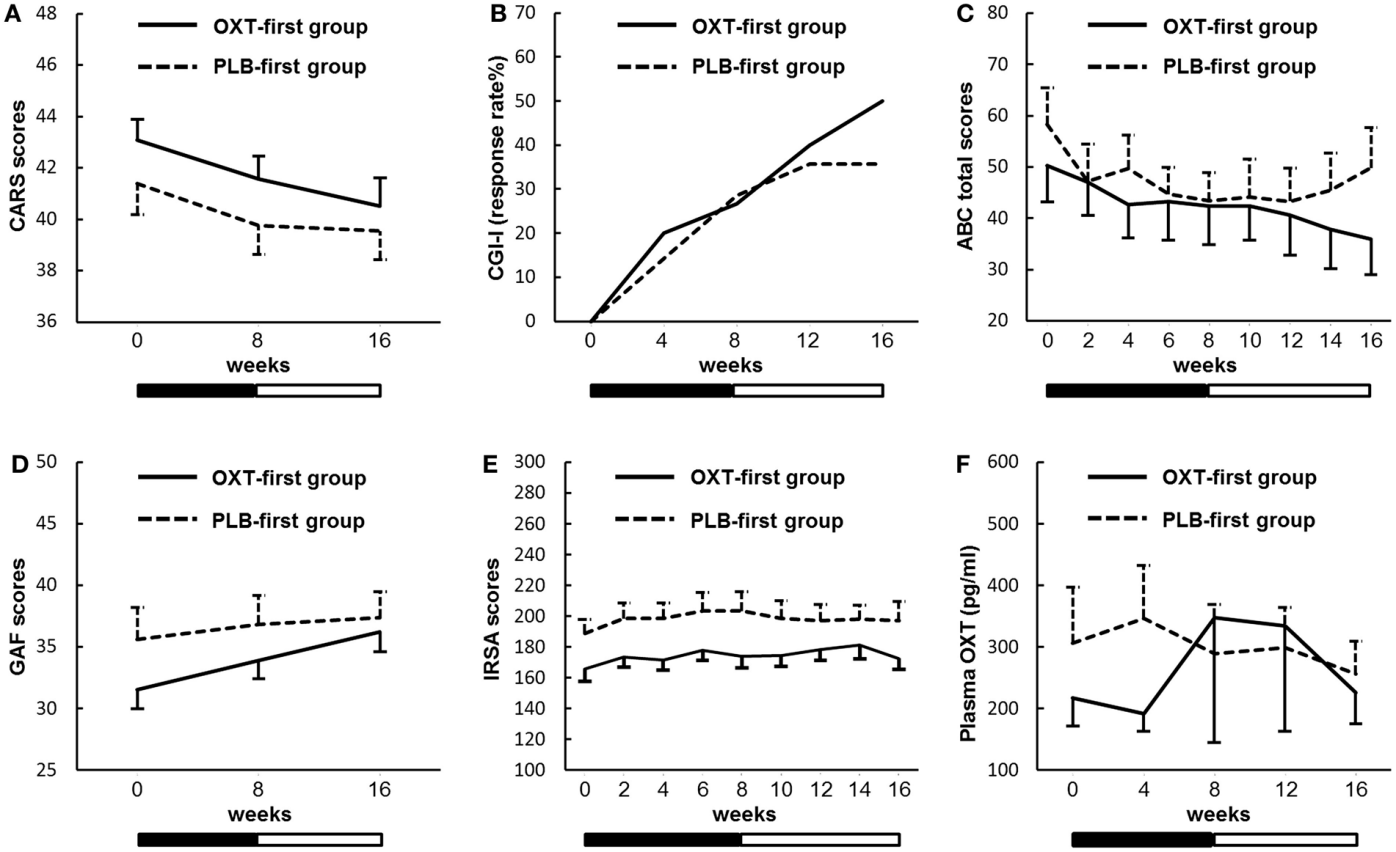
**Sequential changes in the outcome measures**. **(A)** The Childhood Autism Rating Scale (CARS). **(B)** The Clinical Global Impressions – Improvement scale (CGI-I). **(C)** The Aberrant Behavior Checklist (ABC). **(D)** The Global Assessment of Functioning (GAF). **(E)** The Interaction Rating Scale Advanced (IRSA). **(F)** Plasma oxytocin (OXT) concentrations. Solid and dashed lines indicate the OXT-first and placebo (PLB)-first groups. Black and white bars indicate the first treatment period and second treatment period, respectively (8 weeks for each period). Error bars indicate the SEM values. Response rates in the CGI-I show the percentages of participants assessed at 1 (very much improved) or 2 (much improved). There were no significant main effects of the treatment on any of the outcome measures.

There were significant main effects for time and order on the CARS. However, there was no main effect of treatment (Figure 3A; Table S5 in Supplementary Material). Furthermore, there was a significant main effect for time but not for treatment during the first treatment period.

### Secondary Outcome Measures

There were no main effects of treatment on the CGI-I scale (Figure 3B), the ABC (Figure 3C), the GAF (Figure 3D), the IRSA (Figure 3E), or the plasma OXT concentrations (Figure 3F). All of the raw data from these measures are shown in Table S5 in Supplementary Material.

As the IRSA is based on retrospective inspection of behavior, it was possible to analyze patients’ behavior by replaying movies monitored by digital video cameras. No significant difference in frequencies and duration of social interaction, such as eye gaze, expressivity, assertiveness, and responsiveness, was detected between the OXT and placebo arms, suggesting that patients’ behaviors in the playroom are not improved by OXT. Improvement of repetitive behavior or focused stereotypies in the playroom was not obvious after OXT treatment by the medical doctor (in CGI). Therefore, we decided to analyze the patients’ behaviors in detail using the records in the medical charts, according to the protocol.

### Assessment of Social Interactions in Daily Life

**Figure 4:**
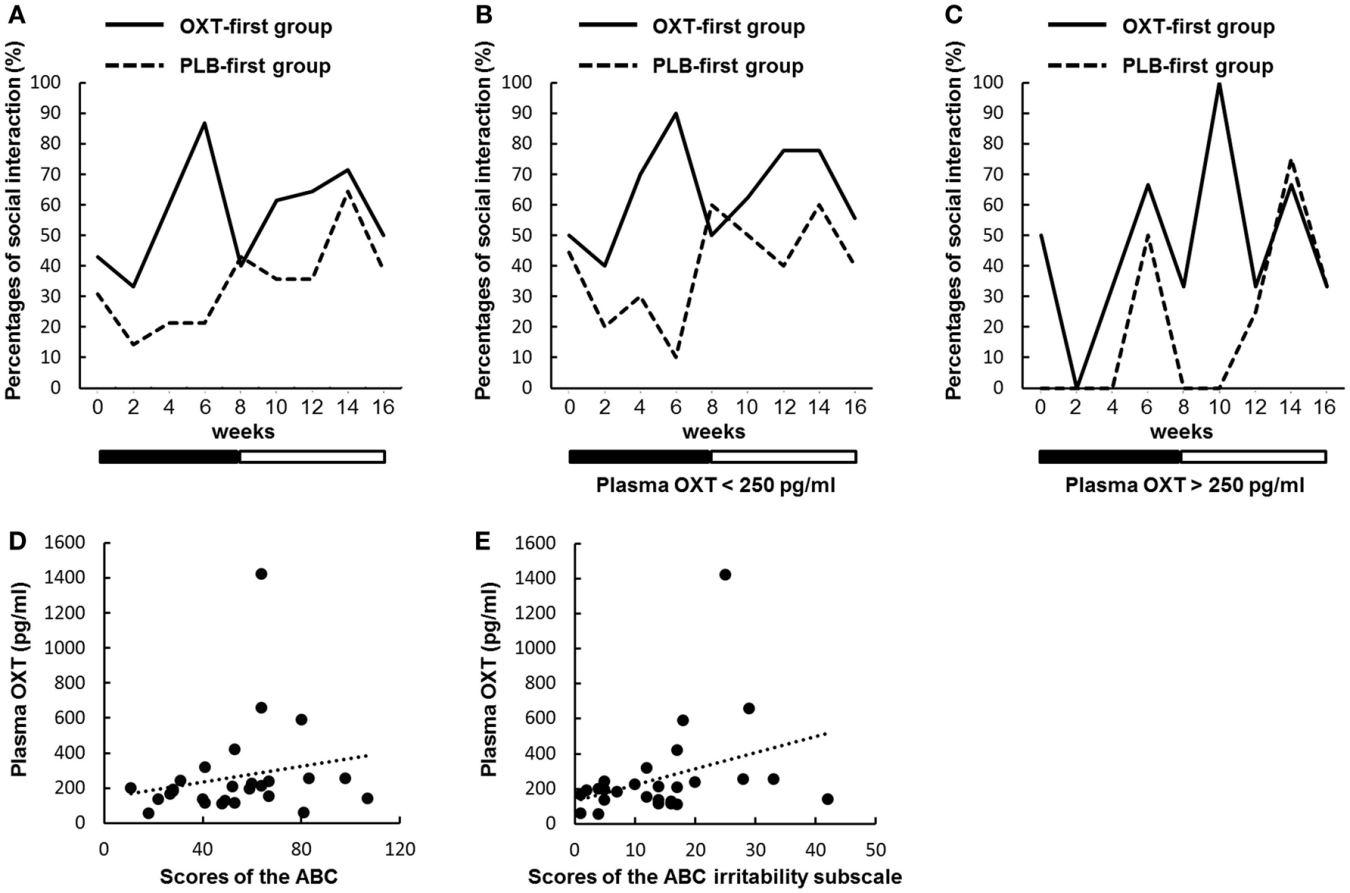
**Sequential changes in the percentages of participants with at least one episode of social interaction**. **(A–C)** Percentages indicate the proportions of all participants who had at least one episode that was regarded as reciprocal social interactions every 2 weeks. Solid and dashed lines indicate the oxytocin (OXT)-first and the placebo (PLB)-first groups. Black and white bars indicate the first treatment period and second treatment period, respectively. **(A)** There were significant main effects for time and order, but not for treatment (*P* = 0.090). However, during the first treatment period, there was a significant main effect of treatment (*P* = 0.029). Moreover, there was a significant interaction between treatment and time (*P* = 0.041). **(B)** Sequential changes of episodes regarded as reciprocal social interactions in participants (*n* = 20) with plasma OXT concentrations below 250 pg/ml. There were significant main effects for time and order, but not for treatment (*P* = 0.109). However, during the first treatment period, there was a significant main effect of treatment (*P* = 0.028). Moreover, there was a significant interaction between treatment and time (*P* = 0.038). **(C)** The same as in B, except participants (*n* = 7) with OXT concentrations over 250 pg/ml. A generalized mixed regression analysis not including ID showed no significant main effect of treatment (*P* = 0.983) during the first treatment period. **(D,E)** Scatter plots portraying the relationships between plasma OXT concentrations and baseline scores on the Aberrant Behavior Checklist (ABC) **(D)** and the ABC irritability subscale **(E)**. The slopes of the dotted linear regression lines indicate a correlation trend [*P* = 0.079 **(D)**] and a significant correlation [*P* = 0.020 **(E)**] in all of the participants.

Table S6 in Supplementary Material lists examples of episodes that were regarded as reciprocal social interactions. We present the changes in the percentages of participants with at least one episode in Figure 4A (see also Table S5 in Supplementary Material). There were significant main effects for time and order, but not for treatment (*P* = 0.090). However, during the first treatment period, there was a significant main effect of treatment (*P* = 0.029). Moreover, there was a significant interaction between treatment and time (*P* = 0.041).

### Plasma Oxytocin Levels

We assessed whether reciprocal social interactions produced by OXT differed in rates, depending on the plasma OXT concentration. The participants were divided into two groups based on the baseline plasma OXT concentration (above or below 250 pg/ml, an appropriate average value for all of the participants) (Figures 4B,C). In the ASD participants with low plasma OXT (below 250 pg/ml, *n* = 20), there were significant main effects for time and order, but not for treatment (*P* = 0.109). However, during the first treatment period, a significant main effect of treatment was observed (*P* = 0.028). Moreover, there was a significant interaction between treatment and time (*P* = 0.038). In the participants with high plasma OXT (above 250 pg/ml, *n* = 7), there was a significant main effect for order, but not for treatment (*P* = 0.805) and time. During the first treatment period, data from seven participants were too small to analyze using a generalized mixed regression model including ID as a covariate. A generalized mixed regression analysis not including ID showed no significant main effect of treatment during the first treatment period (*P* = 0.983).

There was a trend toward a correlation between the baseline OXT concentrations and baseline scores on the ABC (*P* = 0.079; Figure 4D), but not for the CARS, GAF, and IRSA. There were also significant correlations between the baseline OXT concentrations and baseline scores on the ABC irritability subscale (*P* = 0.020; Figure 4E).

## Discussion

To the best of our knowledge, this is the first randomized study of OXT that enrolled male patients with ASD and ID. Although the participants were of a wide range of ages (from 15 to 40 years old) and intellectual levels, the results suggest that long-term clinical trials of intranasal OXT administration are acceptable for ASD individuals with ID. In this study, the outcomes based on conventional measurements demonstrated no significant differences between OXT and the placebo treatments. However, we found that the reciprocal social interactions exhibited by the participants in their daily life, as noticed by their caregivers or in play sessions observed by a researcher, were significantly more frequent during the period of OXT administration than placebo. In addition, lower levels of plasma OXT at baseline predicted significant increases in social interactions, and there were significant correlations between the baseline OXT concentrations and scores on the ABC irritability subscale. False control rates were not taken into consideration because this is the pilot study with a small sample size.

### Differences in Various Assessments

We detected an inconsistency between the results based on the existing scales, e.g., the CARS or IRSA, and a newly designed, but not yet validated, method of assessing real-life social interactions. The negative findings on the former scales could partially be attributed to the low sensitivity of these assessments, which are based on observation, the low dose of OXT or the small sample size. However, the positive findings using the latter method were obtained from behaviors that were unintentionally noticed by evaluators or caregivers. Our results are consistent with the report by Dadds et al. in which a study staff individual could reliably guess the chances of whether participants had received OXT or the placebo, although there were no significant differences in any of the measured outcomes (46). Thus, these results suggest that there may be a measurement that is capable of detecting behavioral changes induced by OXT. In future studies, it will be necessary to standardize the behavior scales observed by family and care givers to further develop this new approach.

The findings in this study in favor of OXT have been obtained by measurements that draw on lists of reciprocal social interactions through various events, both in daily life and in play sessions. This is not surprising because this observation is assessed by bidirectionality, an intrinsic nature of reciprocal social interactions. The theoretical basis for this is that, according to the study by Lombardo and Baron-Cohen (47), social interactions with others imply bidirectional processes. Perceptions of others partly determine how one perceives oneself and perceptions of oneself have an impact on how one perceives others. Furthermore, many studies have indicated a lack or diminution of self-awareness in individuals with ASD (48, 49). Importantly, we know that social interactions with an ASD individual would not be bidirectional for a typically developing individual. In this sense, “social deficits” can indicate the difficulties that typically developing individuals encounter in interacting with ASD individuals. Given that OXT affects self-awareness (50), OXT could produce bidirectional aspects of relationships that emerge not in the whole but in parts of daily life, which seems to be difficult to measure with conventional scales, as indicated in this study.

### Comparison with Recent Clinical Trials

Currently, there are several reports of clinical trials on repeated application of OXT and reviews of these clinical trials. Randomized controlled trials reveal potentially promising findings in measures of emotion and eye gaze, which are impaired early in the course of ASD (51). In contrast, ASD participants who received OXT showed no benefit following treatment on primary or secondary outcomes, although caregivers who believed their wards received OXT report greater improvements (28). Long-term OXT administration results in improved scores on the communication and social interaction domains of the ADOS-G and positive effects on quality of reciprocal communication (52). Over 12 weeks of treatment, several measures of social cognition/function, repetitive behaviors, and anxiety show susceptibility to change (18). In addition, as crosstalk exists between vasopressin and OXT receptors, the OXT effects for ASD patients should be considered with regard to the vasopressin system (53).

### Placebo Effects

The possible efficacy of OXT in the current result may have been masked by placebo effects. Improvements were found in both the OXT and placebo phases when evaluated using the CARS, CGI-I, and ABC (Figures 4A–C). In contrast, the scores on the IRSA, evaluated by independent raters using video images, showed only a few changes (Figure 4D). These effects seem to be artifacts of the placebo, judging from the results of a previous study that found a significant main effect for time and no main effect for treatment (46). The staff and caregivers in the current study seemed to adequately perform their duties. Thus, it was conceivable that the expectation of beneficial effects of OXT under good participant–clinician relationships produced placebo responses (54, 55).

### Plasma OXT Concentrations

We found positive correlations between the plasma OXT concentrations and the baseline severity of aberrant behaviors. This observation is in agreement with the previous result that higher OXT levels were more correlated with developmental delays in individuals with ASD (56). In addition, a significant correlation between the basal OXT levels and the baseline ratings of the Yale–Brown Obsessive-Compulsive Scale has been demonstrated in individuals with obsessive-compulsive disorder (57), which sometimes co-occurs with ASD (58). Altogether, the current study indicates that ASD individuals with lower plasma OXT concentrations may show greater responses to external OXT through nasal administration and suggests that OXT may have modulated novel social interactions. Monitoring OXT concentrations in future trials would be indispensable to improving our understanding of this phenomenon.

Here, we measured the OXT concentration in the unextracted plasma throughout the experiments using the old version of the Assay Designs’ OXT assay kit (currently, Enzo’s EIA kit), as we previously reported (37). Recently, the manufacturer’s protocol recommends performing the assay in fractioned plasma to increase the specificity and sensitivity of the anti-OXT antibody. Thus, the OXT level in the unextracted plasma, which is unmasked by the plausible OXT binding proteins, is usually estimated to be higher by 3- to 10-fold (59, 60). This point should be further explored in future studies.

### Adverse Effects of OXT

Although few adverse events were observed in the study, the seizures that occurred in one participant should be discussed. Anagnostou et al. reported that one participant with stable epilepsy had staring spells 4 weeks following OXT administration and discontinued the intervention (27). However, the anticonvulsive effect of OXT has also been demonstrated (61). Therefore, whether the seizures in our study were caused by OXT remains unclear, although we cannot neglect the possibility of OXT-induced seizures. Future studies are necessary to examine the occurrence of seizures during long-term OXT administration. Closer attention should be paid to ASD in future trials of OXT, especially in patients with ID, because the prevalence of epilepsy has been estimated to be 46.0% in ASD individuals with IQs below 50 (30).

### Limitations

First, the limitations of this study include the standard limitations of the randomized, double-blind, and placebo-controlled crossover study design. Some participants were included who should not have been eligible for participation in ordinary randomized, controlled trials, e.g., subjects receiving psychotropic medications, with comorbid epilepsy or medically serious conditions. Furthermore, we were unable to determine the required sample size in February 2012 when the study was registered. Given the effect size (Cohen’s *d* = 0.57) available now (23), the minimum sample size is estimated to be 82 (parameters: α, 0.05; power, 0.80). Thus, the insignificant difference observed in this study could be attributable to the small sample size of 30.

Second, when we designed this study, we understood that we needed an efficient tool to measure patients’ social interaction at their home. However, because we had no knowledge of any appropriate questionnaires or other methods to assess this parameter, we did not include behavioral analysis at home in the secondary outcomes. Subsequently, we intended to use the medical charts for this aspect, as had been described in the original protocol. Recently, we found such a questionnaire, called the Home Situations Questionnaire for Autism Spectrum Disorder, does exist (62, 63). The current real-life assessment of reciprocal social interactions that has not been adequately validated is thus the exploratory analysis. Given that it is difficult to detect changes caused by treatment through observation, we must use the Home Situations Questionnaire or develop other measurements, bearing in mind the unique characteristics of social interaction in future studies.

Third, it is uncertain whether the treatment drugs were sprayed sufficiently deep into the nasal cavity. Sixteen IUs of OXT may have been a low dose compared with that used in previous studies (i.e., 48 IU). As previous studies have suggested a dose–response phenomenon in the effects of OXT, the negative findings about OXT effects on the primary and secondary endpoints might be attributable to the relatively low dose of OXT used in this study [see Ref. (53)]. However, we chose the dose because we assumed that one spray in each nostril every morning and evening would be easy for participants with ID to perform. Moreover, it was safe to prescribe lower doses of a trial medication, as none of us knew the details of the adverse effects of OXT. None of the participants were reported to be reluctant to receive the treatment. Considering that blood was sampled approximately 6 h or more after spraying in the morning, the plasma OXT concentrations may not have indicated whether the spraying procedures were correctly followed (64–66).

Fourth, we used a single study setting, including the dose and duration of OXT administration, which may not be enough to elicit significant results. Presently, the ideal study conditions have yet to be identified, but we are on the way to contributing to this knowledge base. Specially, no applicable prior studies among ASD patients with ID were identified.

## Conclusion

This pilot study suggests that long-term administration of intranasal OXT would be tolerable for ASD patients with ID under careful supervision for the adverse events reported herein, including seizures. The results should be considered for reference only due to the limitations of this study, although they suggest that a new scale should be designed to assess sociability symptom changes elicited by OXT administration for ASD patients with ID.

## Author Contributions

Conceived and designed the study: TM, HK, YoM, and HH. Performed the study: TM, KT, YS, KM, HidN, YN, and HK. Analyzed the data: TM, KM, HirN, MK, YuM, NT, TA, EN, KA, HH, and SH. Wrote the manuscript: TM, HirN, SY, and HH.

## Conflict of Interest Statement

The authors declare that the research was conducted in the absence of any commercial or financial relationships that could be construed as a potential conflict of interest.
