# Supplementary material for: Oxytocin for Male Subjects with Autism Spectrum Disorder and Comorbid Intellectual Disabilities: A Randomized Pilot Study
Source: Front Psychiatry. 2016 Jan 21;7:2. doi: 10.3389/fpsyt.2016.00002 (PMC4720778; doi:10.3389/fpsyt.2016.00002)
Supplement: Supplementary file 1 [file Table_1.PDF]

Supplementary Table S1

Details of participant characteristics

No	First treatment	Age	Intelligence Quotient	Severity of intellectual disabilities	Psychotropic medications before and during the study	Stable epilepsy	Remarks
2	Oxytocin	25	17	profound	N/A	N/A	
3	Oxytocin	23	Unmeasurable	profound	risperidone	N/A	
4	Oxytocin	26	23	severe	risperidone, sodium valproate	+	
8	Oxytocin	21	Unmeasurable	profound	fluvoxamine, levomepromazine, sodium valproate, risperidone, nitrazepam	N/A	
10	Oxytocin	33	18	profound	haloperidol, levomepromazine, gabapentin, clobazam, phenobarbital, sodium valproatel, flunitrazepam	Attacks had been caused one or two times a year.	
11	Oxytocin	25	Unmeasurable	profound	carbamazepine, sodium valproate, quetiapine	+	
13	Oxytocin	19	Unmeasurable	profound	carbamazepine, olanzapine, quetiapine	N/A	
16	Oxytocin	25	Unmeasurable	profound	quetiapine, hydroxyzine	N/A	
17	Oxytocin	22	Unmeasurable	profound	sodium valproate, zonisamide	+	Discontinued the study
19	Oxytocin	17	13	profound	aripiprazole, ramelteon, flunitrazepam	N/A	
20	Oxytocin	18	Unmeasurable	profound	sodium valproate	N/A	
21	Oxytocin	26	53	mild	olanzapine, paliperidone	N/A	
24	Oxytocin	19	26	moderate	N/A	N/A	
28	Oxytocin	17	24	moderate	N/A	N/A	Intolerable to blood

							drawing
29	Oxytocin	23	Unmeasurable	profound	carbamazepine, risperidone, diazepam	N/A	Intolerable to blood drawing
1	Placebo	32	39	moderate	flunitrazepam	N/A	Provided informed assent
5	Placebo	18	25	severe	olanzapine	N/A	
6	Placebo	21	45	moderate	haloperidol	N/A	Provided informed assent
7	Placebo	18	50	mild	carbamazepine	N/A	Provided informed assent
9	Placebo	25	Unmeasurable	profound	risperidone	N/A	
12	Placebo	21	15	profound	risperidone, sodium valproate, levomepromazine	N/A	
14	Placebo	25	Unmeasurable	profound	sodium valproate, clonazepam	+	
15	Placebo	31	42	moderate	sodium valproate	+	Provided informed assent
18	Placebo	15	59	mild	olanzapine	N/A	
22	Placebo	17	42	moderate	N/A	N/A	
23	Placebo	40	Unmeasurable	profound	N/A	N/A	
25	Placebo	16	44	moderate	N/A	N/A	
26	Placebo	18	15	profound	N/A	N/A	
27	Placebo	17	37	moderate	sodium valproate, olanzapine, quetiapine	+	

N/A, not applicable
