# Supplementary material for: Oxytocin for Male Subjects with Autism Spectrum Disorder and Comorbid Intellectual Disabilities: A Randomized Pilot Study
Source: Front Psychiatry. 2016 Jan 21;7:2. doi: 10.3389/fpsyt.2016.00002 (PMC4720778; doi:10.3389/fpsyt.2016.00002)
Supplement: Supplementary file 2 [file Table_2.PDF]

Supplementary Table S2

Percentages of treatment adherence

		First treatment period				Second treatment period			
No	First treatment	Week 2	Week 4	Week 6	Week 8	Week 10	Week 12	Week 14	Week 16
2	Oxytocin	100	100	100	100	100	100	100	100
3	Oxytocin	100	100	100	100	100	100	100	100
4	Oxytocin	100	100	100	100	100	100	100	100
8	Oxytocin	100	100	100	100	100	96	100	93
10	Oxytocin	100	100	100	100	100	100	96	96
11	Oxytocin	93	100	89	86	82	96	96	93
13	Oxytocin	100	100	100	100	100	100	100	100
16	Oxytocin	96	100	96	71	96	96	100	100
17	Oxytocin	100	100	100	100	100	N/A	N/A	N/A
19	Oxytocin	93	96	93	96	93	100	93	100
20	Oxytocin	100	100	100	100	100	100	100	100
21	Oxytocin	100	100	96	96	100	100	100	100
24	Oxytocin	96	100	96	96	96	89	89	100
28	Oxytocin	100	100	100	100	82	100	100	96
29	Oxytocin	100	100	100	100	100	100	100	100
Mean		98.5	99.7	98	96.3	96.6	98.4	98.1	98.4
1	Placebo	100	100	100	100	100	100	100	100
5	Placebo	100	100	100	100	100	100	100	96
6	Placebo	100	100	100	100	100	100	100	100
7	Placebo	100	100	100	100	100	100	100	96
9	Placebo	100	93	93	93	96	93	89	93
12	Placebo	100	93	100	86	93	100	86	82
14	Placebo	96	100	100	100	100	100	100	96
15	Placebo	100	100	100	100	100	96	100	100
18	Placebo	100	96	100	100	96	96	100	100
22	Placebo	93	100	100	93	100	93	100	100
23	Placebo	100	100	100	100	100	100	100	96
25	Placebo	100	100	100	100	100	100	93	100

26	Placebo	93	100	100	96	100	96	89	100
27	Placebo	100	100	100	100	100	100	100	100
Mean		98.7	98.7	99.5	97.7	98.9	98.1	96.9	97.1

Values indicate percentage. N/A, not available.
