# Supplementary material for: Oxytocin for Male Subjects with Autism Spectrum Disorder and Comorbid Intellectual Disabilities: A Randomized Pilot Study
Source: Front Psychiatry. 2016 Jan 21;7:2. doi: 10.3389/fpsyt.2016.00002 (PMC4720778; doi:10.3389/fpsyt.2016.00002)
Supplement: Supplementary file 3 [file Table_3.PDF]

Supplementary Table S3

Adverse events

	First treatment period		Second treatment period		Post-treatment period	
	OT	PB	PB	OT	First OT	First PB
N	15	14	15	14	15	14
**Behavioral**						
Agitation	3 (20%)	3 (21.4%)	4 (26.7%)	3 (21.4%)	1 (6.7%)	0
Insomnia	2 (13.3%)	0	1 (6.7%)	0	1 (6.7%)	0
Somnolence	2 (13.3%)	0	0	1 (7.1%)	0	0
Attentiveness decreased	0	1 (7.1%)	0	2 (14.3%)	0	0
Mental dullness	2 (13.3%)	0	0	0	0	0
Soliloquy	1 (6.7%)	0	0	1 (7.1%)	1 (6.7%)	0
Polydipsia	0	1 (7.1%)	0	1 (7.1%)	0	0
Hyperkinesia	1 (6.7%)	0	0	0	0	0
Restlessness	0	1 (7.1%)	0	0	0	0
Talkativeness	0	0	0	1(7.1%)	0	0
Trichotillomania	0	0	0	1 (7.1%)	0	0
Decreased activity	0	0	0	0	0	1 (7.1%)
Tic	0	0	0	0	0	1 (7.1%)
**Neurological**						
Headache	1 (6.7%)	0	1 (6.7%)	2 (14.3%)	0	0
Convulsion	1 (6.7%)*	0	1 (6.7%)*	0	1 (6.7)*	1 (7.1%)**
Tremor	0	0	0	1 (7.1%)	0	1 (7.1%)
Muscle spasms	0	0	0	1 (7.1%)	0	0
Hyperacusis	0	0	0	0	0	1 (7.1%)
**Special senses**						

Conjunctival injection	0	1 (7.1%)	0	0	0	0
Keratoconus	0	0	0	0	1 (6.7%)	0
Conjunctivitis	0	0	0	0	0	1 (7.1%)
**Respiratory**						
Rhinorrhea	1 (6.7%)	2 (14.3%)	2 (13.3%)	2 (14.3%)	2 (13.3%)	1 (7.1%)
Nasopharyngitis	2 (13.3%)	2 (14.3%)	1 (6.7%)	1 (7.1%)	0	0
Cough	1 (6.7%)	2 (14.3%)	0	3 (21.4%)	0	0
Bronchitis	1 (6.7%)	0	0	1 (7.1%)	0	0
Upper respiratory inflammation	1 (6.7%)	0	0	0	0	0
Acute sinusitis	0	0	0	1 (7.1%)	0	1 (7.1%)
Epistaxis	0	1 (7.1%)	0	0	0	0
Nasal congestion	0	2 (14.3%)	0	0	0	0
Influenza	0	1 (7.1%)	0	0	0	0
**Cardiovascular**						
Blood pressure increased	0	0	1 (6.7%)	0	0	0
**Gastrointestinal**						
Diarrhoea	4 (26.7%)	1 (7.1%)	3 (20%)	1 (7.1%)	2 (13.3%)	1 (7.1%)
Vomiting	1 (6.7%)	3 (21.4%)	1 (6.7%)	1 (7.1%)	1 (6.7%)	0
Decreased appetite	1 (6.7%)	1 (7.1%)	1 (7.1%)	1 (7.1%)	0	0
Salivary hypersecretion	1 (6.7%)	1 (7.1%)	0	0	0	0
Dental caries	1 (6.7%)	0	0	0	0	0
Acute gastritis	1 (6.7%)	0	0	0	0	0
Perianal abscess	1 (6.7%)	0	0	0	0	0
Abdominal pain	0	0	2 (13.3%)	1 (7.1%)	0	0
Stomatitis	0	1 (7.1%)	0	1 (7.1%)	0	0
Hiccups	0	0	0	1 (7.1%)	0	0
Nausea	0	1 (7.1%)	0	0	0	0
Dental praque	0	1 (7.1%)	0	0	0	0

Toothache	0	1 (7.1%)	0	0	0	0
Faecal incontinence	0	1 (7.1%)	0	0	0	0
**Genitourinary**						
Pollakiuria	0	0	0	2 (14.3%)	1 (6.7%)	0
Polyuria	1 (6.7%)	1 (7.1%)	0	0	0	0
Erectile dysfunction	1 (6.7%)	0	0	0	0	0
Oliguria	1 (6.7%)	0	0	0	0	0
**Dermal**						
Impetigo	2 (13.3%)	0	0	0	0	0
Injury	1 (6.7%)	1 (7.1%)	0	1 (7.1%)	0	0
Eczema	1 (6.7%)	0	0	0	0	0
Rash	1 (6.7%)	0	0	0	0	0
Purpura	1 (6.7%)	0	0	0	0	0
Miliaria	0	1 (7.1%)	0	1 (7.1%)	0	0
Bruising	0	0	0	1 (7.1%)	0	1 (7.1%)
Tinea	0	0	0	1 (7.1%)	0	0
Oedema	0	1 (7.1%)	0	0	0	0
Contact dermatitis	0	0	1 (6.7%)	0	0	0
Pruritis	0	0	1 (6.7%)	0	0	0
Ingrowing nail	0	0	0	0	0	1 (7.1%)
**Musculoskeletal**						
Tenosynovitis	0	0	0	0	1 (6.7%)	0
**General**						
Heat illness	0	1 (7.1%)	0	0	0	0
Cold extremities	0	0	0	0	1 (6.7%)	0
Fever	0	0	0	0	0	1 (7.1%)

*All of the seizures had occurred in one participant (participant number 17 in the Supplementary Table S1).

**We postulated that this seizure might have been due to a failure to take anti-epileptic medication for 2 or 3 days prior to the seizure (participant number 14).
