# Supplementary material for: Oxytocin for Male Subjects with Autism Spectrum Disorder and Comorbid Intellectual Disabilities: A Randomized Pilot Study
Source: Front Psychiatry. 2016 Jan 21;7:2. doi: 10.3389/fpsyt.2016.00002 (PMC4720778; doi:10.3389/fpsyt.2016.00002)
Supplement: Supplementary file 4 [file Table_4.PDF]

Supplementary Table S4 Body weight (kg)

			First treatment period		Second treatment period		Post-treatment period	
No	First treatment	Week 0	Week 4	Week 8	Week 12	Week 16	Week 20	Week 24
2	Oxytocin	66.5	65.5	66	65	65	65.5	64.5
3	Oxytocin	60	58.5	58	57	57	58	56
4	Oxytocin	79.5	80.5	81.5	81	83	83	84
8	Oxytocin	55	54	54	53.5	53.5	54.5	55.5
10	Oxytocin	111	108	107.5	106	106	108	107.5
11	Oxytocin	89	89	89.5	88.5	91	90	90.5
13	Oxytocin	59	59	58.5	59.5	59	58.5	58
16	Oxytocin	66	67	67.5	67	67.5	66.5	66.5
17	Oxytocin	59.5	N/A	58.5	N/A	N/A	N/A	N/A
19	Oxytocin	85	83	83.5	81	82.5	82	84
20	Oxytocin	107	108	108	108	108.5	-	107.5
21	Oxytocin	71	68.5	71.5	68	69	68.5	69.5
24	Oxytocin	58.5	60	60.5	60.5	62	63	62.5
28	Oxytocin	N/A	N/A	N/A	N/A	N/A	N/A	N/A
29	Oxytocin	74	74.5	74	73.5	74	74	77
1	Placebo	80.5	77	73.5	75	73	72.5	72
5	Placebo	49	50	50	50.5	51	52	51.5
6	Placebo	52.5	53	52	53.5	55	55	54.5
7	Placebo	73.5	73	72	70.5	71	72.5	74
9	Placebo	60	60	60	61	62	62	61
12	Placebo	50	49	50	49	48.5	50	50.5
14	Placebo	60	59.5	61	61	61.5	60.5	61.5
15	Placebo	75	76.5	76	78	78.5	78	77
18	Placebo	56	55	54.5	56	56	56	55
22	Placebo	65.5	66.5	65.5	65	62.5	64	66
23	Placebo	70	70	70	70	70	69.5	70
25	Placebo	67	69	67	66	67	68.5	69
26	Placebo	65.5	65	64.5	63	63.5	63.5	64
27	Placebo	79.5	76	74	75	75.5	76	76.5

N/A, not available.

Diastolic blood pressure (mmHg)

			First treatment period		Second treatment period		Post-treatment period	
No	First treatment	Week 0	Week 4	Week 8	Week 12	Week 16	Week 20	Week 24
2	Oxytocin	72	68	66	65	68	65	76
3	Oxytocin	72	70	70	81	64	67	58
4	Oxytocin	78	66	70	78	80	73	73
8	Oxytocin	90	89	86	85	77	82	82
10	Oxytocin	76	75	97	85	72	86	58
11	Oxytocin	93	85	89	62	88	96	94
13	Oxytocin	68	74	65	60	72	62	64
16	Oxytocin	74	79	88	74	86	78	76
17	Oxytocin	82	N/A	68	N/A	N/A	N/A	N/A
19	Oxytocin	66	88	88	72	78	76	68
20	Oxytocin	90	-	86	84	80	68	76
21	Oxytocin	102	84	98	86	88	82	102
24	Oxytocin	64	68	72	62	58	74	70
28	Oxytocin	N/A	N/A	N/A	N/A	N/A	N/A	N/A
29	Oxytocin	N/A	N/A	N/A	68	66	84	78
1	Placebo	90	88	93	108	87	79	95
5	Placebo	58	65	72	55	67	76	65
6	Placebo	62	56	58	64	72	63	64
7	Placebo	63	62	61	62	67	67	74
9	Placebo	74	70	74	91	81	73	86
12	Placebo	89	91	88	84	93	92	82
14	Placebo	65	64	80	71	66	74	68
15	Placebo	93	96	89	88	88	98	102
18	Placebo	78	60	68	54	80	78	70
22	Placebo	78	82	64	64	54	62	74
23	Placebo	78	74	68	72	66	72	72
25	Placebo	80	58	66	78	76	72	74
26	Placebo	62	62	60	54	58	54	58
27	Placebo	60	58	62	66	48	70	68

N/A, not available.

Systolic blood pressure (mmHg)

			First treatment period		Second treatment period		Post-treatment period	
No	First treatment	Week 0	Week 4	Week 8	Week 12	Week 16	Week 20	Week 24
2	Oxytocin	115	114	113	114	108	113	125
3	Oxytocin	125	141	129	126	120	131	111
4	Oxytocin	119	117	123	126	137	117	137
8	Oxytocin	133	126	129	136	123	123	127
10	Oxytocin	133	117	150	144	139	158	154
11	Oxytocin	128	130	107	118	126	126	140
13	Oxytocin	106	115	109	117	109	108	114
16	Oxytocin	118	117	129	94	126	122	118
17	Oxytocin	116	N/A	106	N/A	N/A	N/A	N/A
19	Oxytocin	114	128	124	114	116	104	108
20	Oxytocin	136	N/A	140	154	144	136	132
21	Oxytocin	156	114	146	128	126	116	144
24	Oxytocin	118	130	108	110	114	114	136
28	Oxytocin	N/A	N/A	N/A	N/A	N/A	N/A	N/A
29	Oxytocin	N/A	N/A	N/A	116	116	122	118
1	Placebo	136	127	127	142	127	123	138
5	Placebo	89	113	113	133	111	122	111
6	Placebo	110	98	108	106	112	107	113
7	Placebo	110	115	106	112	119	114	110
9	Placebo	114	118	112	144	129	117	130
12	Placebo	133	138	124	120	141	134	125
14	Placebo	110	111	112	118	114	116	110
15	Placebo	148	135	145	136	136	154	139
18	Placebo	125	106	116	136	128	120	130
22	Placebo	114	138	114	130	128	132	132
23	Placebo	110	108	106	104	110	104	110
25	Placebo	102	118	110	132	118	128	114
26	Placebo	94	90	90	86	88	82	90
27	Placebo	114	112	116	129	118	126	142

N/A, not available.

Pulse rate (per one minute)

			First treatment period		Second treatment period		Post-treatment period	
No	First treatment	Week 0	Week 4	Week 8	Week 12	Week 16	Week 20	Week 24
2	Oxytocin	75	67	70	61	63	60	80
3	Oxytocin	73	85	78	84	75	68	80
4	Oxytocin	82	84	80	74	83	85	82
8	Oxytocin	92	89	75	80	89	97	86
10	Oxytocin	83	105	93	90	83	97	116
11	Oxytocin	97	97	87	97	73	N/A	N/A
13	Oxytocin	79	86	80	75	90	84	88
16	Oxytocin	66	77	67	92	88	76	68
17	Oxytocin	92	-	80	N/A	N/A	N/A	N/A
19	Oxytocin	88	80	92	76	72	68	64
20	Oxytocin	84	-	102	102	90	76	104
21	Oxytocin	80	68	80	84	80	80	88
24	Oxytocin	88	84	84	78	68	80	84
28	Oxytocin	N/A	N/A	N/A	N/A	N/A	N/A	N/A
29	Oxytocin	N/A	N/A	N/A	72	76	90	72
1	Placebo	78	80	82	84	72	80	82
5	Placebo	87	82	80	78	86	70	85
6	Placebo	81	60	56	88	97	86	93
7	Placebo	71	68	68	61	72	86	99
9	Placebo	82	78	76	80	68	83	96
12	Placebo	104	94	85	93	118	92	96
14	Placebo	69	68	90	80	72	108	92
15	Placebo	63	90	82	88	84	92	100
18	Placebo	70	80	98	84	88	108	92
22	Placebo	84	84	72	76	60	76	100
23	Placebo	68	80	72	76	72	76	68
25	Placebo	96	108	84	96	92	84	88
26	Placebo	92	66	72	68	64	76	80
27	Placebo	100	72	78	68	84	68	76

N/A, not available.
