# Supplementary material for: Oxytocin for Male Subjects with Autism Spectrum Disorder and Comorbid Intellectual Disabilities: A Randomized Pilot Study
Source: Front Psychiatry. 2016 Jan 21;7:2. doi: 10.3389/fpsyt.2016.00002 (PMC4720778; doi:10.3389/fpsyt.2016.00002)
Supplement: Supplementary file 5 [file Table_5.PDF]

Supplementary Table S5

Lists of Raw data

Raw data from the Childhood Autism Rating Scale

No			First treatment period	Second treatment period	Post-treatment period
	First treatment	Week 0	Week 8	Week 16	Week 24
2	Oxytocin	43.5	43	43	43.5
3	Oxytocin	45	45	44	45
4	Oxytocin	40.5	40.5	40	40.5
8	Oxytocin	41	39.5	39.5	40
10	Oxytocin	42	40.5	41	40.5
11	Oxytocin	43.5	43.5	40.5	40.5
13	Oxytocin	46	37.5	36	36.5
16	Oxytocin	38	36.5	34	34
17	Oxytocin	43	43	N/A	N/A
19	Oxytocin	44.5	44.5	42	42
20	Oxytocin	49	47.5	49	49
21	Oxytocin	43	43	41	43
24	Oxytocin	46.5	42	44.5	44.5
29	Oxytocin	38	34.5	34.5	35
29	Oxytocin	43	43	38	39
1	Placebo	39.5	41	41	40.5
5	Placebo	46.5	41	43.5	44.5
6	Placebo	37.5	38	37.5	37.5
7	Placebo	33.5	32.5	33	33
9	Placebo	47.5	47.5	46.5	47.5
12	Placebo	50.5	46	47	47
14	Placebo	44	44	41.5	42
15	Placebo	39	38	36	37
18	Placebo	38.5	36.5	36.5	38.5
22	Placebo	41	35	35	37.5

23	Placebo	43	42	38.5	40
25	Placebo	37.5	36.5	37	37.5
26	Placebo	40.5	40.5	40.5	40.5
27	Placebo	41	38	40	41

Higher scores indicate more severe autism spectrum disorder symptoms. N/A, not available.

Raw data from the Clinical Global Impression—Improvement

		First treatment period		Second treatment period		Post-treatment period	
No	First treatment	Week 4	Week 8	Week 12	Week 16	Week 20	Week 24
2	Oxytocin	3	3	3	3	4	4
3	Oxytocin	4	4	3	3	3	4
4	Oxytocin	4	4	3	3	3	4
8	Oxytocin	3	2	2	2	2	3
10	Oxytocin	3	3	4	4	5	4
11	Oxytocin	3	4	2	2	2	2
13	Oxytocin	2	1	1	1	1	1
16	Oxytocin	4	3	2	2	2	2
17	Oxytocin	4	4	4	N/A	N/A	N/A
19	Oxytocin	4	4	3	3	4	4
20	Oxytocin	4	3	4	4	4	4
21	Oxytocin	4	3	3	2	3	4
24	Oxytocin	2	2	4	4	4	4
28	Oxytocin	2	2	2	2	2	3
29	Oxytocin	4	4	2	1	3	3
1	Placebo	4	5	5	5	5	5
5	Placebo	2	1	2	2	3	3

6	Placebo	4	4	4	4	4	4
7	Placebo	4	3	3	4	4	4
9	Placebo	4	4	3	3	3	4
12	Placebo	2	1	1	3	3	3
14	Placebo	4	3	2	2	2	3
15	Placebo	4	3	3	2	3	3
18	Placebo	4	3	3	3	4	4
22	Placebo	4	3	2	2	2	3
23	Placebo	3	3	3	2	2	3
25	Placebo	3	2	2	3	4	4
26	Placebo	3	3	3	3	3	4
27	Placebo	3	2	3	4	4	4

Values indicate as follows: 1, very much improved; 2, much improved; 3, minimally improved; 4, no change; 5, minimally worse; 6, much worse; 7, very much worse. N/A, not available.

Raw data from the Aberrant Behavior Checklist

**Total**												
			First treatment period				Second treatment period				Post-treatment period	
No	First treatment	Week 0	Week 2	Week 4	Week 6	Week 8	Week 10	Week 12	Week 14	Week 16	Week 20	Week 24
2	Oxytocin	59	56	43	39	40	38	36	35	32	33	33
3	Oxytocin	28	24	21	20	20	24	30	25	26	19	31
4	Oxytocin	41	31	34	26	27	34	22	21	19	20	26
8	Oxytocin	64	60	60	52	61	54	36	39	32	41	53
10	Oxytocin	18	16	14	11	11	21	13	19	18	14	10
11	Oxytocin	64	72	49	72	48	53	59	35	54	52	60
13	Oxytocin	40	41	20	26	20	38	25	8	13	38	25
16	Oxytocin	53	66	60	58	53	38	48	46	41	34	27
17	Oxytocin	53	37	47	36	33	39	N/A	N/A	N/A	N/A	N/A
19	Oxytocin	11	13	6	5	6	1	0	1	0	11	1

20	Oxytocin	41	53	59	69	89	74	85	83	82	80	81
21	Oxytocin	81	79	80	75	75	75	75	77	72	85	76
24	Oxytocin	67	38	38	51	46	48	43	50	34	32	41
28	Oxytocin	16	19	16	4	6	4	2	2	5	1	9
29	Oxytocin	119	101	93	104	101	95	95	88	75	70	92
1	Placebo	31	24	30	34	34	26	30	28	31	28	30
5	Placebo	107	106	98	69	78	100	87	96	92	89	96
6	Placebo	80	48	56	33	61	N/A	N/A	N/A	N/A	N/A	N/A
7	Placebo	48	42	37	43	35	23	26	23	45	34	29
9	Placebo	98	77	90	66	75	68	62	56	82	62	56
12	Placebo	52	46	43	44	40	35	49	46	50	47	47
14	Placebo	67	68	62	70	63	53	57	57	60	58	58
15	Placebo	83	76	72	70	55	84	72	74	79	88	89
18	Placebo	28	49	53	60	39	53	52	56	34	82	67
22	Placebo	49	17	25	23	21	21	14	19	19	24	33
23	Placebo	27	24	27	21	23	20	21	16	11	11	20
25	Placebo	60	38	49	44	47	51	48	66	50	68	54
26	Placebo	22	7	15	9	14	14	8	5	10	13	19
27	Placebo	64	39	38	39	22	25	37	48	84	45	44

Irritability

			First treatment period				Second treatment period				Post-treatment period	
No	First treatment	Week 0	Week 2	Week 4	Week 6	Week 8	Week 10	Week 12	Week 14	Week 16	Week 20	Week 24
2	Oxytocin	5	3	3	2	3	3	3	2	3	3	3
3	Oxytocin	2	2	2	3	3	4	4	4	3	0	4
4	Oxytocin	16	14	17	13	12	18	13	11	9	12	14
8	Oxytocin	14	18	15	10	17	14	10	10	10	12	10
10	Oxytocin	4	5	2	3	3	11	5	10	5	5	2
11	Oxytocin	29	31	19	29	11	15	21	14	14	16	16
13	Oxytocin	14	12	5	7	4	12	7	2	2	5	3
16	Oxytocin	17	20	19	17	16	14	16	15	16	14	11
17	Oxytocin	14	7	10	8	4	7	N/A	N/A	N/A	N/A	N/A
19	Oxytocin	4	3	0	1	2	0	0	0	0	5	0
20	Oxytocin	12	16	16	19	22	20	22	23	24	22	24

21	Oxytocin	1	0	0	0	0	0	0	0	0	2	0
24	Oxytocin	12	5	5	8	6	9	9	11	6	5	5
28	Oxytocin	2	3	2	0	0	0	0	0	1	0	0
29	Oxytocin	32	29	25	27	29	25	25	22	17	17	27
1	Placebo	5	4	5	5	6	3	6	7	7	6	9
5	Placebo	42	40	29	23	29	32	31	36	36	37	35
6	Placebo	18	11	13	15	30	N/A	N/A	N/A	N/A	N/A	N/A
7	Placebo	17	13	11	16	12	9	8	11	15	12	11
9	Placebo	33	25	30	25	26	23	23	18	30	22	18
12	Placebo	17	14	13	11	13	4	15	10	11	16	16
14	Placebo	20	20	16	19	17	11	13	15	17	17	15
15	Placebo	28	22	19	20	16	29	22	25	29	29	32
18	Placebo	7	9	9	10	3	10	12	14	0	23	14
22	Placebo	16	9	9	8	7	10	7	7	6	8	6
23	Placebo	1	0	0	0	0	0	1	0	0	0	0
25	Placebo	10	8	8	6	6	14	8	21	10	17	13
26	Placebo	5	2	4	0	0	3	0	0	0	0	0
27	Placebo	25	13	15	14	9	9	15	22	31	19	15
**Lethargy**												
					First treatment period			Second treatment period		Post-treatment period		
No	First treatment	Week 0	Week 2	Week 4	Week 6	Week 8	Week 10	Week 12	Week 14	Week 16	Week 20	Week 24
2	Oxytocin	27	27	20	21	19	19	19	19	17	17	17
3	Oxytocin	2	2	1	1	4	5	7	5	7	4	7
4	Oxytocin	7	4	6	6	6	4	2	2	1	2	3
8	Oxytocin	14	10	11	13	10	5	1	2	0	3	10
10	Oxytocin	6	5	5	2	2	2	2	2	4	2	2
11	Oxytocin	10	10	6	22	10	14	15	6	19	10	13
13	Oxytocin	3	3	5	6	2	2	0	0	1	0	0
16	Oxytocin	14	20	12	15	13	10	12	11	10	7	10
17	Oxytocin	12	7	10	7	10	11	N/A	N/A	N/A	N/A	N/A
19	Oxytocin	3	5	4	1	1	0	0	1	0	2	1
20	Oxytocin	12	16	16	19	22	20	22	23	24	22	24

21	Oxytocin	40	41	39	38	41	40	42	39	40	43	42
24	Oxytocin	14	7	9	9	8	10	9	8	7	7	9
28	Oxytocin	0	4	6	2	3	2	1	0	1	0	0
29	Oxytocin	28	22	21	26	23	22	25	25	23	16	22
1	Placebo	8	6	10	9	8	6	9	5	6	7	8
5	Placebo	19	13	14	16	9	16	9	10	11	9	15
6	Placebo	24	7	9	2	4	N/A	N/A	N/A	N/A	N/A	N/A
7	Placebo	8	7	6	5	6	1	2	2	5	5	2
9	Placebo	12	7	11	10	5	6	6	5	10	4	6
12	Placebo	19	16	21	20	18	15	19	18	17	15	15
14	Placebo	18	18	18	21	17	16	16	16	16	17	16
15	Placebo	13	11	10	11	4	10	13	12	7	13	10
18	Placebo	0	3	4	1	1	1	2	1	1	6	2
22	Placebo	11	1	2	3	2	1	0	3	3	6	9
23	Placebo	16	16	17	12	11	10	13	9	6	6	12
25	Placebo	8	4	6	5	6	3	5	7	3	5	3
26	Placebo	10	5	7	6	7	6	6	3	6	6	9
27	Placebo	11	6	5	3	3	3	6	5	16	7	6

**Stereotypic behavior**												
			First treatment period				Second treatment period				Post-treatment period	
No	First treatment	Week 0	Week 2	Week 4	Week 6	Week 8	Week 10	Week 12	Week 14	Week 16	Week 20	Week 24
2	Oxytocin	10	10	8	7	7	7	7	8	5	7	7
3	Oxytocin	11	9	10	10	8	9	11	10	8	10	13
4	Oxytocin	2	3	3	1	2	2	1	3	3	2	2
8	Oxytocin	7	8	7	7	7	9	7	7	7	7	8
10	Oxytocin	0	0	0	0	0	0	0	0	0	0	0
11	Oxytocin	0	2	4	2	4	3	3	2	2	2	4
13	Oxytocin	6	6	4	5	8	4	8	4	6	8	8
16	Oxytocin	6	9	11	8	7	4	6	6	6	5	0
17	Oxytocin	14	13	13	13	12	13	N/A	N/A	N/A	N/A	N/A
19	Oxytocin	1	0	0	1	1	1	0	0	0	2	0
20	Oxytocin	5	3	8	7	9	7	9	9	9	6	8

21	Oxytocin	18	18	19	21	20	20	20	21	19	21	21
24	Oxytocin	10	6	4	6	5	4	3	2	2	1	2
28	Oxytocin	0	1	1	0	1	0	0	0	0	0	0
29	Oxytocin	15	15	15	15	15	15	15	15	13	13	15
1	Placebo	2	2	1	2	1	1	1	1	1	1	1
5	Placebo	4	14	14	5	11	15	12	14	12	13	12
6	Placebo	2	9	8	4	4	N/A	N/A	N/A	N/A	N/A	N/A
7	Placebo	2	0	0	1	1	0	0	0	4	1	0
9	Placebo	19	19	18	11	17	17	15	14	15	14	13
12	Placebo	6	9	3	6	4	11	8	12	10	8	8
14	Placebo	9	7	6	7	7	7	7	7	7	7	7
15	Placebo	3	1	2	1	2	1	1	2	1	2	1
18	Placebo	7	14	14	18	16	21	20	21	18	21	21
22	Placebo	2	0	1	1	1	0	1	1	1	0	1
23	Placebo	0	0	1	1	2	0	0	1	0	0	0
25	Placebo	16	9	12	12	13	12	14	13	14	15	13
26	Placebo	0	0	0	0	0	0	0	0	0	0	0
27	Placebo	1	1	0	0	0	1	1	2	2	2	1

**Hyperactivity**												
			First treatment period				Second treatment period				Post-treatment period	
No	First treatment	Week 0	Week 2	Week 4	Week 6	Week 8	Week 10	Week 12	Week 14	Week 16	Week 20	Week 24
2	Oxytocin	9	9	5	4	4	3	2	0	2	2	1
3	Oxytocin	11	9	6	4	4	5	6	4	6	3	5
4	Oxytocin	10	7	5	3	4	6	3	3	3	2	5
8	Oxytocin	24	19	22	18	23	20	14	16	11	15	20
10	Oxytocin	2	0	1	0	0	2	0	1	3	1	0
11	Oxytocin	21	22	14	15	14	14	11	6	13	18	21
13	Oxytocin	9	10	1	3	3	11	3	0	0	15	5
16	Oxytocin	11	10	10	11	12	3	6	8	3	2	0
17	Oxytocin	13	10	14	8	7	8	N/A	N/A	N/A	N/A	N/A
19	Oxytocin	3	3	1	2	2	0	0	0	0	2	0
20	Oxytocin	8	16	17	20	27	23	24	26	23	24	24

21	Oxytocin	21	20	22	16	13	15	13	14	13	19	13
24	Oxytocin	26	17	18	24	23	22	19	25	18	18	22
28	Oxytocin	5	3	1	0	0	0	0	0	0	0	1
29	Oxytocin	35	26	23	27	25	24	21	17	16	15	19
1	Placebo	7	5	6	8	9	8	4	8	9	6	4
5	Placebo	30	27	29	16	20	26	23	25	22	20	23
6	Placebo	28	14	19	9	19	N/A	N/A	N/A	N/A	N/A	N/A
7	Placebo	12	15	13	15	9	6	9	6	14	11	10
9	Placebo	34	26	31	20	27	22	18	19	27	22	19
12	Placebo	8	7	6	7	5	5	7	6	12	8	8
14	Placebo	16	17	16	16	16	15	16	15	16	13	16
15	Placebo	27	30	29	26	21	32	24	23	30	32	34
18	Placebo	6	12	19	20	10	12	8	8	7	23	21
22	Placebo	12	3	9	8	4	5	2	4	4	4	9
23	Placebo	3	1	3	2	2	2	1	1	0	0	2
25	Placebo	16	10	14	12	13	14	11	18	15	21	17
26	Placebo	5	0	3	1	7	5	1	1	2	1	2
27	Placebo	20	14	13	14	5	7	10	10	24	8	16

**Inappropriate speech**												
			First treatment period				Second treatment period				Post-treatment period	
No	First treatment	Week 0	Week 2	Week 4	Week 6	Week 8	Week 10	Week 12	Week 14	Week 16	Week 20	Week 24
2	Oxytocin	8	7	7	5	7	6	5	6	5	4	5
3	Oxytocin	2	2	2	2	1	1	2	2	2	2	2
4	Oxytocin	6	3	3	3	3	4	3	2	3	2	2
8	Oxytocin	5	5	5	4	4	6	4	4	4	4	5
10	Oxytocin	6	6	6	6	6	6	6	6	6	6	6
11	Oxytocin	4	7	6	4	9	7	9	7	6	6	6
13	Oxytocin	8	10	5	5	3	9	7	2	4	10	9
16	Oxytocin	5	7	8	7	5	7	8	6	6	6	6
17	Oxytocin	0	0	0	0	0	0	N/A	N/A	N/A	N/A	N/A
19	Oxytocin	0	2	1	0	0	0	0	0	0	0	0
20	Oxytocin	5	6	6	6	7	7	8	6	7	7	8

21	Oxytocin	1	0	0	0	1	0	0	3	0	0	0
24	Oxytocin	5	3	2	4	4	3	3	4	1	1	3
28	Oxytocin	9	8	6	2	2	2	1	2	3	1	8
29	Oxytocin	9	9	9	9	9	9	9	9	6	9	9
1	Placebo	9	7	8	10	10	8	10	7	8	8	8
5	Placebo	12	12	12	9	9	11	12	11	11	10	11
6	Placebo	8	7	7	3	4	N/A	N/A	N/A	N/A	N/A	N/A
7	Placebo	9	7	7	6	7	7	7	4	7	5	6
9	Placebo	0	0	0	0	0	0	0	0	0	0	0
12	Placebo	2	0	0	0	0	0	0	0	0	0	0
14	Placebo	4	6	6	7	6	4	5	4	4	4	4
15	Placebo	12	12	12	12	12	12	12	12	12	12	12
18	Placebo	8	11	7	11	9	9	10	12	8	9	9
22	Placebo	8	4	4	3	7	5	4	4	5	6	8
23	Placebo	7	7	6	6	8	8	6	5	5	5	6
25	Placebo	10	7	9	9	9	8	10	7	8	10	8
26	Placebo	2	0	1	2	0	0	1	1	2	6	8
27	Placebo	7	5	5	8	5	5	5	9	11	9	6

Higher scores indicate worse behaviors. N/A, not available.

Raw data from the Interaction Rating Scale Advanced

			First treatment period				Second treatment period				Post-treatment period	
No	First treatment	Week 0	Week 2	Week 4	Week 6	Week 8	Week 10	Week 12	Week 14	Week 16	Week 20	Week 24
2	Oxytocin	156	176	180	197	201	170	164	155	N/A	180	116
3	Oxytocin	131	131	142	137	136	140	135	139	143	140	140
4	Oxytocin	N/A	191	169	221	148	172	156	217	176	176	199
8	Oxytocin	152	152	159	162	168	145	139	150	138	140	140
10	Oxytocin	138	194	146	196	197	209	207	203	189	208	197
11	Oxytocin	218	216	216	215	218	216	222	N/A	155	220	173

13	Oxytocin	118	138	149	158	162	191	188	219	170	167	170
16	Oxytocin	168	161	159	164	166	168	165	163	162	162	162
17	Oxytocin	178	188	188	188	188	N/A	N/A	N/A	N/A	N/A	N/A
19	Oxytocin	187	187	212	191	193	N/A	193	188	201	188	188
20	Oxytocin	191	160	163	154	163	149	182	158	160	154	159
21	Oxytocin	197	200	200	197	215	203	215	213	209	209	209
24	Oxytocin	146	141	145	138	119	161	159	159	167	152	167
28	Oxytocin	190	194	182	182	176	193	202	227	224	200	203
29	Oxytocin	148	170	161	167	157	149	172	163	151	164	164
1	Placebo	236	248	248	275	275	266	185	207	212	209	232
5	Placebo	139	164	161	146	159	166	180	156	166	182	182
6	Placebo	197	219	212	N/A	N/A	N/A	N/A	N/A	N/A	N/A	N/A
7	Placebo	202	224	211	221	229	205	233	220	235	243	222
9	Placebo	152	155	161	161	137	156	151	158	137	137	139
12	Placebo	151	176	146	146	137	134	128	154	96	134	136
14	Placebo	186	150	185	185	185	206	187	187	206	231	207
15	Placebo	256	249	246	246	254	238	243	240	245	249	249
18	Placebo	197	219	227	227	227	171	201	202	209	178	197
22	Placebo	202	202	199	213	214	217	214	214	217	218	233
23	Placebo	176	172	178	209	191	206	191	191	191	191	182
25	Placebo	209	258	261	261	261	258	269	267	270	272	272
26	Placebo	162	162	162	159	181	162	181	178	178	178	178
27	Placebo	182	179	185	194	194	199	199	199	199	204	199

Higher scores indicate higher levels of social competence. N/A, not available.

Raw plasma oxytocin concentration data (pg/ml)

			First treatment period		Second treatment period		Post-treatment period	
No	First treatment	Week 0	Week 4	Week 8	Week 12	Week 16	Week 20	Week 24
2	Oxytocin	197	147	141	197	203	211	198
3	Oxytocin	191	197	143	295	236	271	248

4	Oxytocin	117	95	54	100	105	113	114
8	Oxytocin	212	202	183	219	310	317	269
10	Oxytocin	54	60	90	52	42	51	92
11	Oxytocin	656	432	2774	2357	520	571	1742
13	Oxytocin	136	210	194	114	169	381	131
16	Oxytocin	419	144	93	55	68	191	97
17	Oxytocin	114	169	146	146	N/A	N/A	N/A
19	Oxytocin	202	172	116	99	135	64	99
20	Oxytocin	317	349	287	437	613	332	N/A
21	Oxytocin	59	106	130	118	116	124	139
24	Oxytocin	155	205	174	159	200	163	191
28	Oxytocin	N/A	N/A	N/A	N/A	N/A	N/A	N/A
29	Oxytocin	N/A	N/A	N/A	N/A	N/A	N/A	N/A
1	Placebo	244	250	243	292	265	320	261
5	Placebo	139	133	193	146	131	151	147
6	Placebo	589	625	511	648	633	937	905
7	Placebo	109	179	98	115	58	96	133
9	Placebo	253	265	217	197	236	295	74
12	Placebo	207	408	303	307	356	261	381
14	Placebo	238	337	247	241	160	303	234
15	Placebo	254	216	148	128	69	167	189
18	Placebo	183	241	265	205	245	208	202
22	Placebo	126	196	183	205	183	145	112
23	Placebo	166	152	152	138	141	174	97
25	Placebo	225	253	93	298	225	215	183
26	Placebo	135	230	138	236	145	135	225
27	Placebo	1420	1372	1259	1024	738	654	579

N/A, not available.

Raw data from the Global Assessment of Functioning

No	First treatment		First treatment period	Second treatment period	Post-treatment period
		Week 0	Week 8	Week 16	Week 24
2	Oxytocin	31	31	31	31
3	Oxytocin	34	34	35	34
4	Oxytocin	33	33	33	33
8	Oxytocin	34	36	36	35
10	Oxytocin	34	34	34	34
11	Oxytocin	28	28	32	32
13	Oxytocin	21	39	40	40
16	Oxytocin	38	39	42	42
17	Oxytocin	34	34	N/A	N/A
19	Oxytocin	21	21	38	38
20	Oxytocin	34	37	34	34
21	Oxytocin	38	38	39	38
24	Oxytocin	21	24	22	22
28	Oxytocin	34	43	43	41
29	Oxytocin	38	38	48	44
1	Placebo	39	38	38	38
5	Placebo	17	31	31	21
6	Placebo	40	21	28	28
7	Placebo	50	50	50	50
9	Placebo	21	21	21	21
12	Placebo	21	34	31	31
14	Placebo	31	31	33	32
15	Placebo	39	39	40	40
18	Placebo	40	43	43	40
22	Placebo	42	43	43	43
23	Placebo	38	39	40	39
25	Placebo	43	46	46	43
26	Placebo	43	43	43	43
27	Placebo	35	37	36	35

Higher ratings indicate better functioning in the social, occupational and psychological domains.

N/A, not available.

Raw data from an exploratory analysis

			First treatment period				Second treatment period			
No	First treatment	Week 0	Week 2	Week 4	Week 6	Week 8	Week 10	Week 12	Week 14	Week 16
2	Oxytocin	-	+	+	+	-	+	+	-	-
3	Oxytocin	+	-	+	+	-	+	+	+	+
4	Oxytocin	-	-	+	+	-	-	+	+	+
8	Oxytocin	+	-	-	+	+	-	+	+	+
10	Oxytocin	-	+	-	+	+	+	+	+	-
11	Oxytocin	N/A	-	-	+	-	+	-	+	-
13	Oxytocin	-	-	+	+	+	+	-	-	-
16	Oxytocin	+	-	+	+	+	+	+	+	+
17	Oxytocin	+	+	+	+	-	N/A	N/A	N/A	N/A
19	Oxytocin	+	-	-	+	-	N/A	+	+	+
20	Oxytocin	-	-	-	-	-	+	-	-	-
21	Oxytocin	+	+	+	+	+	+	+	+	+
24	Oxytocin	-	-	+	-	+	-	-	+	-
28	Oxytocin	-	-	-	+	-	-	-	+	+
29	Oxytocin	-	+	+	+	-	-	+	-	-
1	Placebo	-	-	-	-	-	-	-	-	-
5	Placebo	-	-	+	-	-	-	-	-	-
6	Placebo	-	-	-	-	-	-	-	-	N/A
7	Placebo	-	-	-	-	-	-	-	+	-
9	Placebo	-	-	-	-	-	-	-	+	-
12	Placebo	N/A	-	-	+	+	+	-	+	-
14	Placebo	-	+	+	-	+	+	+	+	+
15	Placebo	-	-	-	+	-	-	+	+	+
18	Placebo	+	-	-	-	+	-	+	-	-
22	Placebo	+	-	-	-	-	+	+	+	+
23	Placebo	+	-	+	-	+	+	+	+	+

25	Placebo	+	-	-	-	+	-	-	+	+
26	Placebo	-	+	-	-	+	+	-	-	-
27	Placebo	-	-	-	+	-	-	-	+	-

+, one or more episodes regarded as reciprocal social interaction; -, no episode; N/A, not available.
