# Supplementary material for: Oxytocin for Male Subjects with Autism Spectrum Disorder and Comorbid Intellectual Disabilities: A Randomized Pilot Study
Source: Front Psychiatry. 2016 Jan 21;7:2. doi: 10.3389/fpsyt.2016.00002 (PMC4720778; doi:10.3389/fpsyt.2016.00002)
Supplement: Supplementary file 6 [file Table_6.PDF]

Supplementary Table S6. Examples of episodes regarded as social interaction

This table shows parts of the descriptions of participants' behaviors obtained from the play sessions and interview sessions in the medical chart. Underlined phrases were blindly regarded as social interactions by two co-authors (NT and YM). A, B and C, the oxytocin arm; D, the placebo arm. See Supplementary Table S1 with regard to details of the participants.

A Participant nember 2: At the end of the sixth week of the first treatment period

Play session	Interview session
As always, he lied down on the examination table while holding a Lego block. When I sat at his feet, he stared at me for quite a long time. He sometimes glanced upward when I returned the gaze. However, at other times he did not maintain eye contact. He worked with enthusiasm on a slightly difficult jigsaw puzzle given to him in the second half of the play session.	His mother said the following: "My sister's family lives nearby and they visit two or three times a month. When they visited on May 17, I felt that my son interrupted my conversations with my sister more often than before."<br>"When my son is alone with me, he do not initiate conversation very often; however, this has become somewhat more frequent."<br>"There have been no other changes."

B Participant nember 8: At the end of the sixth week of the first treatment period

Play session	Interview session
His handling of a mosaic puzzle have become more skillful. His expressions are also more firm than before. However, he does not maintain eye contact as always. When walking down the hallway in the hospital, he did not show usual restricted interests in doorknobs. When the session has come to an end, he did not comply by saying "no" until the mosaic puzzle was complete, and did not want to leave. Previously, he would respond immediately and leave, even if the puzzle was unfinished.	His mother said the following: "My son was less lethargic and did not wander around as much as that seen previously."<br>"The staff at the vocational aid center said that there has been no change."<br>"My son sometimes has drawn pictures of *Anpanman* as always in home. Recently, *Anpanman*'s expression appears to soften."<br>"He did not write very often in the past but has been writing a lot lately."

C Participant nember 16: At the end of the sixth week of the first treatment period

Play session	Interview session
He began to assemble a mosaic puzzle as usual. Previously, he would not go home until the puzzle was finished; however, today he surprisingly stopped when told to stop and began to put the puzzle away.	His parents said the following: "We do not think that there has been any change."<br>"Although, there was one thing, he always want to buy "*Donbei*" (instant udon noodles) whenever we go to a convenience store. However, the other day, when we told him, "Stop buying that," he gave up. We have not experienced anything like this before."<br>"Recent entries in the correspondence book from the vocational aid center indicated that he seemed to be more motivated while at work."

D Participant nember 27: At the end of the sixth week of the first treatment period

Play session	Interview session
He sat cross-legged on the floor in front of	His mother said the following: "When I

the bookcase and began to see a picture book as always. When I asked him “How are you?” or “Did you eat a lunch?” from behind, he replied some words after about 10 seconds. I thought these responses had not appeared previously.	have picked up my son at the school, he recently has begun to speak, e.g., that he had done his best today or that he had acted violently. Previously, only when I asked him, he replied.”<br>“Self-injurious behaviors have lessened a little”<br>“Restricted behaviors were as usual.”
