# Supplementary material for: Oxytocin for Male Subjects with Autism Spectrum Disorder and Comorbid Intellectual Disabilities: A Randomized Pilot Study
Source: Front Psychiatry. 2016 Jan 21;7:2. doi: 10.3389/fpsyt.2016.00002 (PMC4720778; doi:10.3389/fpsyt.2016.00002)
Supplement: Supplementary file 8 [file Data_Sheet_2.PDF]

Supplementary Information S2

26 January 2012

Study protocol (Important notes)

An exploratory, randomized, double-blind, placebo-controlled, crossover study of oxytocin in individuals with autism spectrum disorders and comorbid intellectual disabilities

This study will be conducted according to the Declaration of Helsinki and the Ethical Guidelines for Clinical Studies by the Ministry of Health, Labour and Welfare of Japan.

Principal investigator: Toshio Munesue

Department of Child and Adolescent Psychiatry

Kanazawa University Hospital

Background

Introduction

Autistic disorder, Asperger disorder and pervasive developmental disorder not otherwise specified are all described in the section on pervasive developmental disorders in the Diagnostic and Statistical Manual of Mental Disorders, fourth edition, text revision (DSM-IV-TR) [1]. These conditions are thought to resemble one another in their symptomatology and are therefore referred to generally as autism spectrum disorders (ASD).

ASD is a syndrome that involves innate deficits of interpersonal exchanges, i.e., social deficits, as a core symptom. There are no fundamental therapies for ASD. Its prevalence has been estimated to be as high as approximately 1% [2].

It is difficult for individuals with ASD to communicate, either verbally or non-verbally, and to work collaboratively. Social deficits cause serious maladaptations within human society, which is based on interpersonal exchanges.

Repetitive behaviors are another core symptom in individuals with ASD. For example, affected individuals may show stereotyped behaviors such as striking an object rhythmically for a prolonged period. Other rituals include going to bed every night

only after indulging in a fixed series of nonsensical acts.

Intelligence levels vary widely among individuals with ASD, ranging from profound disabilities to high levels of intelligence. Individuals with ASD who have intellectual disabilities and their caregivers are often troubled by behaviors such as hyperkinesis, screaming, temper tantrums, self-harming, prolonged repetitive acts and violence. These behaviors may be serious burdens for caregivers from patients' infancy to through adulthood [3].

Because there is no fundamental therapy for ASD directed at the actual disease mechanisms, treatments focus on the symptoms. Behavioral therapies are intended to identify how patients with ASD live in society. Pharmacological therapies, which target irritability as manifested by, for example, temper tantrums or self-harming, include antipsychotics, which have been confirmed to be effective to some extent in randomized controlled trials [4]. However, no treatments have yet shown effectiveness for the core symptoms of social deficits and repetitive behaviors [5]. Several drugs, such as omega-3 fatty acids [6] and tetrahydrobiopterin [7], have been investigated as potential therapies, but the results were unsuccessful.

Biological background of this study

The cell bodies of oxytocin-producing neurons are located in the hypothalamus, and their axons terminate in the posterior pituitary. Oxytocin (OXT), a peptide hormone secreted into the circulatory systems from the axon terminals in the posterior pituitary, plays important roles in uterine contraction at delivery and lactation during the postpartum period.

OXT is also secreted into the brain from the dendrites of the OXT-producing neurons in the hypothalamus [8]. Although the half-life of OXT in the blood stream is relatively short (approximately 2 minutes), the half-life in the brain is approximately 20 minutes [9], suggesting that OXT may diffuse widely throughout the brain. Because neurons with OXT receptors can also secrete OXT, the amount of OXT is thought to increase rapidly in the brain owing to positive feedback.

The functions of OXT in the brain have gradually been clarified. OXT gene knock-out mice and OXT-receptor gene knock-out mice lack the ability to remember partner mice [10, 11]. CD38, a transmembrane glycoprotein, plays an essential role in the secretion of OXT in the brain [12]; CD38 gene knock-out mice also show an impaired ability to remember partner mice [12]. These results suggest that OXT may influence social interactions between individuals.

In humans, many studies have been conducted to investigate the functions of OXT

due to experiments using functional magnetic resonance imaging technics or psychological tasks in typically developing individuals [13-25]. For example, responses to the sound of an infant crying showed reduced activation in the amygdala (the neural center for anxiety) and increased activation in the insula and inferior frontal gyrus (regions related to empathy) after intranasal OXT administration to female subjects [13]. Subjects administered OXT intranasally perceived face stimuli as more trustworthy and attractive than subjects administered placebo [19]. These results suggest that OXT may play a favorable role in enhancing social cognition. However, other studies have produced the contrary findings [26-30]. For example, when subjects administered OXT intranasally gained more money than their opponents, they expressed increased gloating to the opponents. In contrast, when they gained less money than their opponents, they expressed increased envy to the opponent [27]. Typically developing subjects with high ASD traits showed increased social cognition after being administered OXT as compared with placebo. By contrast, subjects with low ASD traits failed to show any differences in the performance of a social cognition task between the two conditions [29]. Therefore, OXT may exhibit distinct effect on the basis of experimental conditions and subjects' characteristics.

Randomized controlled trials of OXT in individuals with ASD

To date, 4 randomized, double-blind, placebo-controlled studies of short-term OXT administration in ASD individuals have produced favorable results in terms of improvements in social deficits and repetitive behaviors [31-34]. However, there are currently no findings related to long-term OXT administration. According to public databases, such as the University Hospital Medical Information Network (UMIN) Clinical Trials Registry and ClinicalTrials.gov, 10 randomized controlled trials on long-term OXT administration are currently registered. Table 1 lists 4 of these trials which, like our planned study, seek to investigate long-term administration of OXY to individuals with ASD.

Table 1 Randomized controlled trials of oxytocin in individuals with autism spectrum disorders

Sponsor	Date of registration	Age (years)	Gender	Sample size	Duration of OXT administration
Fukui University	8 March 2011	≥ 15	Both	60	12 weeks
Montefiore Medical Center	15 December 2010	18 - 55	Both	34	8 weeks
University of Illinois	22 November 2010	12 - 18	Both	-	12 weeks
University of North Carolina, Chapel Hill	28 February 2011	3 - 17	Both	30	16 weeks

The main objective of our study is to evaluate the therapeutic and adverse effects

of intranasal OXT administration in adolescents and adults with ASD and comorbid intellectual disabilities.

Methods

Design

This is an exploratory, randomized, double-blind, placebo-controlled, crossover study.

The baseline period (2 weeks)

* Prior to the start of the study, informed consent, confirmation of eligibility, and assessments will be obtained.
* Enrollment and assignment will be carried out.

The first treatment period (8 weeks)

* Participants will receive either 8 international units (IU) of OXT twice per day or a matching placebo twice per day administered in the form of an intranasal spray.
* Participants will visit the Kanazawa University Hospital every two weeks to undergo medical examination, symptom assessment and harm assessment.

The second treatment period (8 weeks)

* After crossover, participants will receive intranasal administration of the other treatment drug and will visit the hospital every two weeks to undergo the same examinations and assessments carried out during the first treatment period.

The post-treatment period (8 weeks)

* Participants will visit Kanazawa University Hospital every four weeks to undergo the same examinations and assessments.

Eligible participants

Selection of participants will be based on the following inclusion and exclusion criteria.

Inclusion criteria;

* Diagnosis of ASD based on the DSM-IV [1], using the Diagnostic Interview for Social and Communication Disorders [35].
* Age from 15 to 45 years old
* Male

* Intelligence quotient < 75 based on the Tanaka-Binet Intelligence Scale
* Ability to tolerate blood sampling

The eligible participants are restricted to males because OXT normally promotes nursing. If OXT is administered to females, it may cause untoward effects including contraction of the smooth muscle in the mammary glands or the uterus. According to the UMIN-Clinical Trials Registry and ClinicalTrials.gov, several clinical trials on OXT have included female participants (Table 1). However, other studies have suggested that there are differences in ASD symptoms [36] or distinct responses to OXT administration between males and females [37].

Exclusion criteria

* Concomitant or previous history of allergy to OXT
* Previous history of traumatic brain injury with loss of consciousness for more than 5 min
* Concomitant or previous history of substance dependence
* History of water intoxication or severe polydipsia
* Presence of common severe polydipsia

Informed consent

The principal investigator will obtain written informed consent from the participants or their legally acceptable representatives after fully explaining this study, using an information document that clearly describes the study.

Enrollment and assignment

Enrollment

After obtaining written informed consent from each participant, the principal investigator will record each participant's name and the day of consent in the participant identification record. The records will be numbered consecutively from 1 to 30. The participant enrollment document and identification records will be submitted to the Clinical Research Center of Kanazawa University Hospital. The Clinical Research Center will provide the investigator with a treatment assignment number for each participant it confirms. During the course of the study, this number will be associated with the drug treatment prescriptions.

Assignment

A single staff member of the Clinical Research Center will prepare the table with

consecutive numbers from 1 to 30, the treatment assignment number and the name of the randomly assigned treatment drug (OXT or placebo). When the staff member obtains the participant enrollment document from the investigators, only the treatment assignment number will be communicated back to the investigators. The staff member will safeguard the table during the study and will not disclose it to others until the day of disclosure of the assignment.

Treatment drugs

Oxytocin

Brand name: Syntocinon (5 ml per bottle)

Manufacturer: Novartis Pharma, S.A.S. (France)

Sales agency: Defiante Farmacéutia, S.A. (Portugal)

Efficacy classification: Promotion of lactation

Mechanism of action: Binding to mammary OXT receptors, leading to the contraction of smooth muscle.

Indication: Promotion of nursing

Usage: Intranasal spray 5 minutes before lactation

Contraindication: None

Main results of clinical use: No relevance to this study

Adverse effects: None

Drug interaction: Unknown

Instructions for clinical use: Store at 2-8 °C

Placebo

The placebo (5ml per bottle), to be prepared in the pharmacy of Kanazawa University Hospital, will consist of the same ingredients of Syntocinon except OXT: chlorobutanol (12.5 mg), *p*-hydroxybenzoic acid methyl ester (1.3mg), *p*-hydroxybenzoic acid propyl ester (0.7 mg), anhydrous sodium dihydrogen phosphate (28 mg), disodium hydrogenorthophosphate (14 mg), 84-87% glycerin (0.5ml), anhydrous citric acid (14.4 mg) and distilled water.

Similarities between the treatment drugs

Syntocinon will be transferred from the original bottle into a sterile test nasal spray bottle (Oono Co., Ltd., Tokyo, Japan), and the placebo will be stored in an identical bottle.

Treatments

The treatment periods of both OXT and the placebo will be 8 weeks. The dose of OXT per day corresponds to 16 IU. Most previous studies used only a single dose of 24 IU of OXT in typically developing adults. All of the previous clinical trials in individuals with ASD also used a single dose of 24 IU of OXT. The clinical trials registered in the UMIN-Clinical Trials Registry or at ClinicalTrials.gov set the following doses of OXT per day: 8-24 IU in the Oosaka University study, 16 or 32 IU in the Fukui University study, 48 IU in the Montefiore Medical Center study, 24 IU in the University of Illinois study and 24 or 32 IU in the University of North Carolina at Chapel Hill study.

Some caregivers have reported administering OXT nasal spray, obtained over the counter, to their children with ASD, with favorable results [38]. The doses in those cases were either 8 or 16 IU per day. The dose of 16 IU in this study is slightly lower than doses administered in other studies, but we chose it because the instruction to spray 4 puffs (16 IU) per day (one puff to each nostril every morning and evening) is clear and easy to understand.

Concomitant therapies

No new treatments targeting various symptoms of ASD will be allowed. Any current

treatments will be continued without alteration, if possible.

Drug therapies that target various symptoms of diseases other than ASD will be allowed. If exacerbations of ASD symptoms or possible adverse events, such as agitation or self-harm, occur in a participant, he may withdraw from this study if he requires psychotropic medications.

Examination and assessments

Treatment adherence

Treatment adherence will be determined using a treatment diary in which each caregiver will record the time of spraying in the morning and evening.

Blood pressure, pulse rate and body weight

These parameters will be measured every 4 weeks during the study.

Blood analysis, urinalysis and electroencephalography

These examinations will be conducted every 4 weeks. The blood analysis will include blood count (WBC, RBC, Hct, Hb and Plt), electrolytes (Na, K and Cl), renal function (BUN and Cr), liver function (AST, ALT, Al-P, LDH, CPK and T-Bil), serum

osmolality, plasma arginine vasopressin concentrations and plasma OXT concentrations. Blood sampling for the DNA analyses described below will be conducted once during the study. All blood collection will be performed between 2 and 5 pm, and the participants will be instructed to refrain from eating two hours before blood sampling. Urine osmolality will be measured in addition to routine urinalysis.

Assessment of harm

Adverse events will be reported by caregivers at every visit using a questionnaire that was developed for this study. The definitions of adverse events were compiled using the Medical Dictionary for Regulatory Activities.

Assessments of participants' symptoms and behaviors

The severity of ASD symptoms will be assessed by the principal investigator every 8 weeks using the Childhood Autism Rating Scale (CARS, [39]).

The Clinical Global Impression—Improvement (CGI-I) scale will be scored by the principal investigator every 4 weeks.

Aberrant behaviors will be assessed by each caregiver every 2 weeks using the Aberrant Behavior Checklist (ABC, [40]).

The Global Assessment of Functioning (GAF) scale will be scored by the principal investigator every 8 weeks.

Plasma OXT concentrations will be assayed by an independent technician.

The genotyping of fragile X syndrome-related genes or other ASD-related genes will be conducted following approval by the Human Genome/Gene Analysis Research Ethics Committee of Kanazawa University (number 272, 28 March 2012 for the fragile X syndrome-related genes; number 213, 16 March 2010 for ASD-related genes).

Because the participants in this study will have intellectual disabilities, they may be unable to verbalize their inner experiences. Accordingly, we will assess their symptoms by observing their behaviors. Prior to the study, a psychiatrist (the principal investigator) will hold biweekly 20-minute play sessions with each participant to observe their characteristic behaviors. After the study begins, each biweekly visit will include both a 20-minute play session and an interview session with the caregivers to determine any changes in behaviors. Each play session will be recorded by two video cameras set up diagonally in the playroom, and the recordings will be assessed by trained evaluators (who are blind to the treatment groups) using the Interaction Rating Scale Advanced (IRSA) [41].

Endpoint

Primary endpoint

Changes in the scores will be evaluated using the CARS.

Secondary endpoints

Changes in the scores will be evaluated according to the ABC, the CGI-I, the GAF and the IRSA, and changes in the plasma OXT concentrations will also be assessed.

Expected results

Advantages

The potential advantages of OXT treatment are unknown, because no randomized controlled trials on long-term administration of OXT to individuals with ASD have yet been published.

Disadvantages

A systematic review article on the adverse effects of the short-term (but not long-term) administration of OXT to healthy adults reported no serious adverse effects [42]. Three case reports on the long-term use or over-dose of oxytocin have been

published. In one, a 55-year-old man received 12 IU per day of intranasally administered OXT to treat obsessive-compulsive disorder for 4 weeks. During the treatment, he complained of auditory hallucinations, persecutory delusions and memory disturbances. Laboratory examinations revealed hyponatremia and low serum osmolality [43]. In another case, a 33-year-old woman who given birth 4 months earlier presented at the hospital because of generalized convulsions and altered consciousness. She was diagnosed with water intoxication due to polydipsia and oxytocin overdose [44]. Finally, a 28-year-old woman who had given birth 5 months earlier continued to use OXT for lactation. She was admitted to the hospital because of lethargy, low-grade fever and myalgia of the lower extremities. While receiving an infusion of saline, she drank water and continued to take OXT. She suffered from generalized convulsions 2 days after admission, and laboratory examinations revealed hyponatremia and low serum osmolality [45]. In these cases, the antidiuretic effects of OXT appear to have induced water intoxication or hyponatremia. Individuals with ASD tend to drink large quantities of water [46]. Accordingly, we set a history of severe polydipsia as an exclusion criterion.

Sample size

Whether long-term administration of OXT is effective in alleviating symptoms of ASD remains unknown owing to the lack of clinical trials. No information of on the effect size is currently available; accordingly, a target sample size cannot be calculated. We set a sample size of 30 in this study on the basis of other randomized clinical trials registered in the UMIN Clinical Trials Registry or at ClinicalTrials.gov: 34 subjects in the Montefiore Medical Center study and 30 subjects in the University of North Carolina at Chapel Hill study (Table 1).

Statistical analysis

An independent statistician who is otherwise not involved in this study will analyze the results using a generalized mixed regression model.

Registration of this study

Before starting this study, we will register the protocol with the UMIN Clinical Trials Registry.

Funding

This study is funded by “Integrated Research on Neuropsychiatric Disorders” under

the Strategic Research Program for Brain Sciences by the Ministry of Education, Culture, Sports, Science and Technology of Japan.

Investigators for this study

Principal investigator:

Toshio Munesue[1]

Co-investigators:

Haruhiro Higashida[1], Yoshio Minabe[2], Shoichi Koizumi[1], Hiroyuki Nakamura[3], Manabu Oi[1], Shigeru Yokoyama[1], Yo Niida[4], Mitsuru Kikuchi[4], Noriyoshi Takeuchi[1], Yui Miura[1]

[1] Research Center for Child Mental Development, Kanazawa University, Kanazawa

[2] Department of Neuropsychiatry, Kanazawa University Hospital, Kanazawa

[3] Department of Environmental and Preventive Medicine, Kanazawa University, Kanazawa

[4] Department of Child and Adolescent Psychiatry, Kanazawa University Hospital, Kanazawa

Corresponding investigator:

Toshio Munesue, MD PhD

Research Center for Child Mental Development, Kanazawa University

13-1 Takara-machi, Kanazawa, Ishikawa, 9208640 Japan

e-mail: munesue@med.kanazawa-u.ac.jp

References

1. American Psychiatric Association (2000) Diagnostic and Statistical Manual of Mental Disorders Fourth Edition Text Revision. American Psychiatric Association, Washington, D. C., 2000.
2. Baird G, Simonoff E, Pickles A, Chandler S, Loucas T, et al. (1998) Prevalence of disorders of the autism spectrum in a population cohort of children in South Thames: the Special Needs and Autism Project (SNAP). Lancet 368: 210-215.
3. Lecavalier L (2006) Behavioral and emotional problems in young people with pervasive developmental disorders: relative prevalence, effects of subject characteristics, and empirical classification. J Autism Dev Disord 36: 1101-1114.
4. McCracken JT, McGough J, Shah B, Cronin P, Hong D, et al. (2002) Risperidone in children with autism and serious behavioral problems. N Engl J Med 347:

314-21.

5. Williams K, Wheeler DM, Silove N, Hazell P (2010) Selective serotonin reuptake inhibitors (SSRIs) for autism spectrum disorder (ASD). Cochrane Database Syst Rev CD004677.

6. Bent S, Bertoglio K, Ashwood P, Bostrom A, Hendren RL (2011) A pilot randomized controlled trial of omega-3 fatty acids for autism spectrum disorder. J Autism Dev Disord 41: 545-554.

7. Frye RE, Huffman LC, Elliott GR (2010) Tetrahydrobiopterin as a novel therapeutic intervention for autism. Neurotherapeutics 7: 241-249.

8. Ludwig M, Leng G (2006) Dendritic peptide release and peptide dependent behaviours. Nat Rev Neurosci 7: 126-136.

9. Mens WBJ, Witter A, van Wimersma-Greidanus TB (1983) Penetration of neurophypophyseal hormones from plasma into cerebrospinal fluid (CSF): half-times of disappearance of these neuropeptides from CSF. Brain Res 262: 143-149.

10. Ferguson JN, Young LJ, Hearn EF, Matzuk MM, Insel TR, et al. (2000) Social amnesia in mice lacking the oxytocin gene. Nature Genet 25: 284-288.

11. Takayanagi Y, Yoshida M, Bielsky IF, Ross HE, Kawamata M, et al. (2005)

Pervasive social deficits, but normal parturition, in oxytocin receptor-deficient mice. Proc Natl Acad Sci USA 102: 16096-16101.

12. Jin D, Liu HX, Hirai H, Torashima T, Nagai T, et al. (2007) CD38 is critical for social behaviour by regulating oxytocin secretion. Nature 446: 41-45.

13. Riem MM, Bakermans-Kranenburg MJ, Pieper S, Tops M, Boksem MA, et al. (2011) Oxytocin modulates amygdala, insula, and inferior frontal gyrus responses to infant crying: a randomized controlled trial. Biol Psychiatry 70: 291-297.

14. Domes G, lischke A, berger C, Grossmann A, Hauenstein K, et al. (2010) Effects of intranasal oxytocin on emotional face processing in women. Psychoneuroendocrinology 35: 83-93.

15. Gamer M, Zurowski B, Buchel C (2010) Different amygdala subregions mediate valence-related and attentional effects of oxytocin in humans. Proc Natl Acad Sci U S A 107: 9400-9405.

16. Kirsch P, Eesslinger X, Chen Q, Mier D, Lis S, et al. (2005) Oxytocin modulates neural circuitry for social cognition and fear in humans. J Neurosci 25: 11489-11493.

17. Fischer-Shofty M, Shamay-Tsoory SG, Harari H, Levkovitz Y (2010) The effect of

intranasal administration of oxytocin on fear recognition. Neuropsychologia, 48: 179-184.

18. Domes G, Heinrichs M, Michel A, Berger C, Herpertz SC (2007) Oxytocin improves "mind-reading" in humans. Biol Psychiatry 61: 731-733.

19. Theodoridou A, Rowe AC, Penton-Voak IS, Rogers PJ (2009) Oxytocin and social perception: oxytocin increases perceived facial trustworthiness and attractiveness. Horm Behav 56: 128-132.

20. Evans S, Shergill SS, Averbeck BB (2010) Oxytocin decreases aversion to angry faces in an associative learning task. Neuropsychopharmacology 35: 2502-2509.

21. Guastella AJ, Mitchell PB, Dadds MR (2008) Oxytocin increases gaze to the eye region of human faces. Biol Psychiatry 63: 3-5.

22. Guastella AJ, Mitchell PB, Mathews F (2008) Oxytocin enhances the encoding of positive social memories in humans. Biol Psychiatry 64: 256-258.

23. Zak PJ, Stanton AA, Ahmadi S (2007) Oxytocin increases generosity in humans. PLoS One 2: e1128.

24. Kosfeld M, Heinrichs M, Zak PJ, Fischbacher U, Fehr E (2005) Oxytocin increases trust in humans. Nature 435: 673-676.

25. Mikolajczak M, Pinon N, Lane A, de Timary P, Luminet O (2010) Oxytocin not only

increases trust when money is at stake, but olso when confidential information is in the balance. Biol Psychiatry 85: 182-184.

26. Declerck CH, Boone C, Kiyonari T (2010) Oxytocin and cooperation under conditions of uncertainty: the modulating role of incentives and social information. Horm Behav 57: 368-374.

27. Shamay-Tsoory SG, Fischer M, Dvash J, Harari H, Perach-Bloom N, et al. (2009) Intranasal administration of oxytocin increases envy and schadenfreude (gloating). Biol Psychiatry 66: 864-870.

28. de Dreu CKW, Greer LL, van Kleef GA, Shalvi S, Handgraaf MJJ (2011) Oxytocin promotes human ethnocentrism. Proc Natl Acad Sci U S A 108: 1262-1266.

29. Bartz JA, Zaki J, Bolger N, Hollander E, Ludwig NN, et al. (2010) Oxytocin selectively improves empathic accuracy. Psychol Sci 21: 1426-1428.

30. Luminet O, Grynberg D, Ruzette N, Mikolajczak M (2011) Personality-dependent effects of oxytocin: greater social benefits for high alexithymia scores. Biol Psychol 87: 401-406.

31. Hollander E, Novotny S, Hanratty M, Yaffe R, DeCaria CM, et al. (2003) Oxytocin infusion reduces repetitive behaviors in adults with autistic and Asperger's disorders. Neuropsychopharmacology 28: 193-198.

32. Hollander E, Bartz J, Chaplin W, Phillips A, Sumner J, et al. (2007) Oxytocin increases retention of social cognition in autism. Biol Psychiatry 61: 498-503.

33. Andari E, Duhamel JR, Zalla T, Herbrecht E, Leboyer M, et al. (2010) Promoting social behavior with oxytocin in high-functioning autism spectrum disorders. Proc Natl Acad Sci U S A 107: 4389-4394.

34. Guastella AJ, Einfeld SL, Gray KM, Rinehart NJ, Tonge BJ, et al. (210) Intranasal oxytocin improves emotion recognition for youth with autism spectrum disorders. Biol Psychiatry 67: 692-694.

35. Wing L, Leekam SR, Libby SJ, Gould J, Larcombe M (2002) The Diagnostic Interview for Social and Communication Disorders: background, inter-rater reliability and clinical use. J Child Psychol Psychiatry 43: 307-325.

36. Rivet TT, Matson JL (2011) Review of gender differences in core symptomatology in autism spectrum disorders. Res Autism Spectr Disord 5: 957-976.

37. Carter CS (2007) Sex differences in oxytocin and vasopressin: implications for autism spectrum disorders? Behav Brain Res 176: 170-186.

38. Munesue T, Yokoyama S, Nakamura K, Anitha A, Yamada K, et al. (2010) Two genetic variants of CD38 in subjects with autism spectrum disorder and controls. Neurosci Res 67: 181-191.

39. Schopler E, Reichler RJ, Renner BR (1986) The Childhood Autism Rating Scale. Irvington Publisher, Inc., New York.

40. Aman MG, Singh NN (1994) Aberrant Behavior Checklist. Slosson Educational Publication, Inc., New York.

41. Anme T, Watanabe T, Tokutake K, Tomisaki E, Mochizuki Y, et al. (2011) A pilot study of social competence assessment using Interaction Rating Scale Advanced(IRSA). Pediatrics 2011: 272913.

42. MacDonald E, Dadds MR, Brennan JL, Williams K, Levy F, et al. (2011) A review of safety, side-effects and subjective reactions to intranasal oxytocin in human research. Psychoneuroendocrinology 36: 1114-1126.

43. Ansseau M, Legros JJ, Mormont C, Cerfontaine JL, Papart P, et al. (1987) Intranasal oxytocin in obsessive-compulsive disorder. Psychoneuroendocrinology 12: 231-236.

44. Mayer-Hubner B (1996) Pseudotumor cerebri from intranasal oxytocin and excessive fluid intake. Lancet 347: 623.

45. Seifer DB, Sandberg EC, Ueland K, Sladen RN (1985) Water intoxication and hyponatremic encephalopathy from the use of an oxytocin nasal spray. J Reprod Med 30: 225-228.

46. Terai K, Munesue T, Hiratani M (1999) Excessive water drinking behavior in autism. Brain Dev 21: 103-106.
