# Supplementary material for: Oxytocin for Male Subjects with Autism Spectrum Disorder and Comorbid Intellectual Disabilities: A Randomized Pilot Study
Source: Front Psychiatry. 2016 Jan 21;7:2. doi: 10.3389/fpsyt.2016.00002 (PMC4720778; doi:10.3389/fpsyt.2016.00002)
Supplement: Supplementary file 9 [file Data_Sheet_3.PDF]

Supplementary Information S3

Informed assent form for intellectually disabled persons

Assent document

Explanation to ______________

This document explains what tests you need to do at the hospital and what you need to do at home.

If there is anything you do not understand in this document, please ask questions.

1. What you need to do at the hospital

☐ Please come to the hospital 12 times.

☐ We will measure your body weight 7 times.

☐ We will measure your blood pressure 7 times.

☐ We will collect your blood 8 times. It will not hurt.

☐ We will collect your urine 7 times.

☐ You will have a test called electrocardiogram 7 times. When you have this test, we will attach things that look like bandages to your chest. They will not hurt.

☐ We will take videos of you 12 times.

2. What you need to do at home.

Please ask your father or mother to spray the drug into your right nostril and left nostril in the morning after you eat breakfast. Start from the fourteenth day from your first visit to the hospital. This will not hurt.

Please ask your father or mother to spray the drug into your right nostril and left nostril again in the evening after you eat dinner. This will not hurt.

☐ Please continue this for 112 days.

After that, all you need to do is come to the hospital twice, and then you will be done.

	1st time	2nd time	3rd time	4th time	5th time	6th time	7th time	8th time	9th time	10th time	11th time	12th time
**Body weight**	○			○		○		○		○	○	○
**Blood pressure**	○			○		○		○		○	○	○
**Electrocardiogram**	○			○		○		○		○	○	○
**Blood**	○	○		○		○		○		○	○	○
**Urine**	○			○		○		○		○	○	○
**Video recording**	○	○	○	○	○	○	○	○	○	○	○	○

○ Indicates things you are going to do.

Mine

At the hospital,

☐ **We will measure your body weight 7 times.**

☐ **We will measure your blood pressure 7 times.**

☐ **We will collect your blood 8 times. This will not hurt.**

☐ **We will collect your urine 7 times.**

☐ **You will have a test called electrocardiogram 7 times.**

☐ **We will take videos of you 12 times.**

At home,

Your father or mother will spray the drug into your nostrils.
You will get the spray for 112 days.

☐ **I will go to the hospital 12 times.**

☐ **I will ask my father or mother to spray the**

drug into my nostrils at home. It will not hurt.
Me

Year Month Day

Name ______________________________

Doctor who explained

Year Month Day

Name ______________________________

Doctor's

At the hospital,

☐ **We will measure your body weight 7 times.**

☐ **We will measure your blood pressure 7 times.**

☐ **We will collect your blood 8 times. It will not hurt.**

☐ **We will collect your urine 7 times.**

☐ **You will have a test called electrocardiogram 7 times.**

☐ **We will take videos of you 12 times.**

At home,

Your father or mother will spray the drug into your nostrils.

You will get the spray for 112 days

☐ **I will go to the hospital 12 times**

☐ **I will ask my father or mother to spray the**

drug into my nostrils. It will not hurt.

Me

Year Month Day

Name ______________________________

Doctor who explained

Year Month Day

Name ______________________________
