# Supplementary material for: Oxytocin for Male Subjects with Autism Spectrum Disorder and Comorbid Intellectual Disabilities: A Randomized Pilot Study
Source: Front Psychiatry. 2016 Jan 21;7:2. doi: 10.3389/fpsyt.2016.00002 (PMC4720778; doi:10.3389/fpsyt.2016.00002)
Supplement: Supplementary file 10 [file Data_Sheet_4.PDF]

# Supplementary Information S4

## Adverse events questionnaire

## Symptoms noted in the participant

**Please tick in the box □ (□) if any of the following symptoms or diseases was noted in the patient to date.**

Form completed on: year month day
Name of the child:
Form completed by: Relationship ( )

[Infectious disease]
□ Pharyngitis
□ Pneumonia
□ Otitis media
□ Influenza
□ Rhinitis
□ Tonsillitis
□ Conjunctivitis
□ Other ( )

[Nutrition-related]
□ Decreased appetite
□ Increased appetite

[Mental]
□ Insomnia
□ Excitement
□ Irritability
□ Restlessness
□ Lack of energy
□ Hallucination
□ Rambling story
□ Soliloquy
□ Allusion to suicide
□ Intentional self-injury
□ Suicide attempt
□ Other ( )

[Nervous system]
□ Hand tremor
□ Trembling of the body
□ Drowsiness
□ Staggering
□ Drooling
□ Numbness
□ Lack of facial expression
□ Strange pronunciation
□ Spasm
□ Muscle stiffness
□ Reduced motion
□ Distraction
□ Headache
□ Other (        )

[Eyes]
□ Strange eye movement
□ Spasm of the eyelid
□ Reduced visual acuity
□ Blurred vision
□ Excessive lacrimation
□ Dry eye
□ Other (        )

[Ears]
□ Tinnitus
□ Other (        )

[Heart]
□ Rapid pulse
□ Slow pulse
□ Arrhythmia
□ Palpitation
□ Other (        )

[Blood vessel]
□ Dizziness on standing up
□ Cold hands and feet

□ Other (          )

[Breathing]

□ Nasal stiffness

□ Difficulty in breathing

□ Coughing

□ Runny nose

□ Loss of voice

□ Hyperventilation

□ Other (          )

[Gastrointestinal]

□ Constipation

□ Diarrhea

□ Nausea

□ Vomiting

□ Difficulty in swallowing

□ Dry mouth

□ Abdominal pain

□ Toothache

□ Fecal incontinence

□ Stomatitis

□ Other (          )

[Skin]

□ Excessive sweating

□ Rash

□ Itchiness of skin

□ Eczema

□ Hair loss

□ Dry skin

□ Other (          )

[Muscle and bone]

□ Muscle pain

□ Twisted neck

□ Stiffness of joint

□ Back pain

□ Pain in hands and feet

□ Other (          )

[Urinary tract]
□ Increased urine output
□ Reduced urine output
□ Pain when urinating
□ Increased frequency of urination
□ Reduced frequency of urination
□ Urinary incontinence
□ Other (          )

[Reproductive]
□ Failure of erection
□ Failure of ejaculation
□ Other (          )

* Please describe anything other than the above that has happened to the patient.
(1) ______________________________
(2) ______________________________
(3) ______________________________
(4) ______________________________

* Please list all the hospital visits, if any.

(1) Hospital name:
Symptom:
Disease name:
Drug prescribed:

(2) Hospital name:
Symptom:
Disease name:
Drug prescribed:

(3) Hospital name:
Symptom:
Disease name:
Drug prescribed:

(4) Hospital name:
Symptom:
Disease name:
Drug prescribed:

**Thank you for completing the form.**
**We will appreciate it if you could take the trouble to recheck for any omissions.**
