# Supplementary figures and images for: Oxytocin for Male Subjects with Autism Spectrum Disorder and Comorbid Intellectual Disabilities: A Randomized Pilot Study
Source: Front Psychiatry. 2016 Jan 21;7:2. doi: 10.3389/fpsyt.2016.00002 (PMC4720778; doi:10.3389/fpsyt.2016.00002)

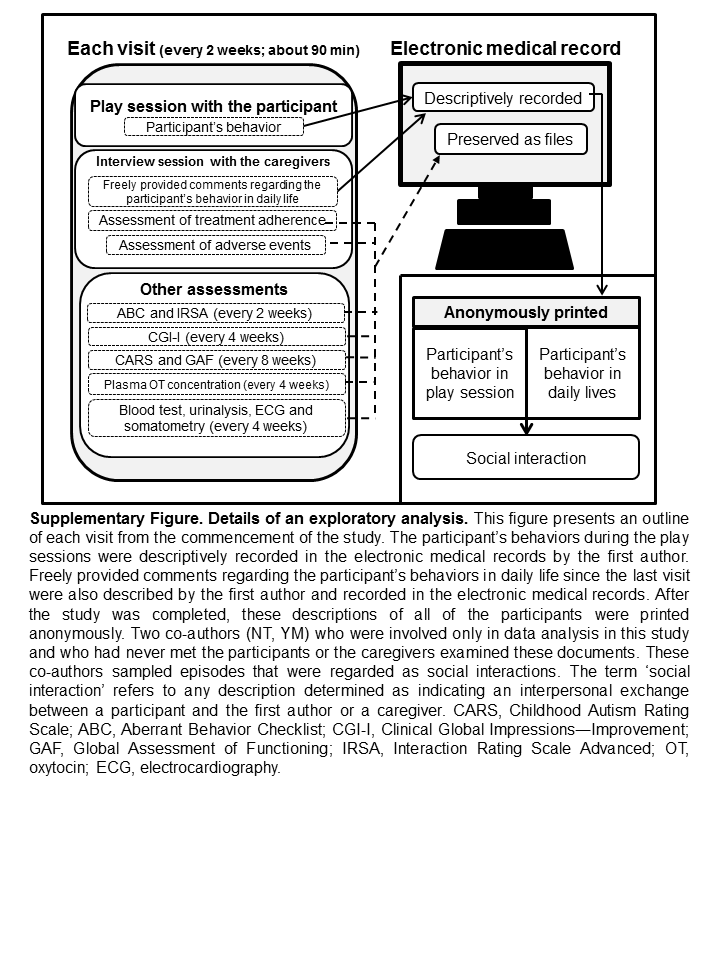

Supplement: Supplementary file 11 [file Image_1.TIF]
